# Unveiling spatial complexity in solid tumor immune microenvironments through multiplexed imaging

**DOI:** 10.3389/fimmu.2024.1383932

**Published:** 2024-03-19

**Authors:** Sophia Scheuermann, Beate Kristmann, Fabienne Engelmann, Alice Nuernbergk, David Scheuermann, Marie Koloseus, Tayeb Abed, Wiebke Solass, Christian M. Seitz

**Affiliations:** ^1^ Department of Haematology, Oncology, Gastroenterology, Nephrology, Rheumatology, University Children’s Hospital Tuebingen, Tuebingen, Germany; ^2^ iFIT Cluster of Excellence EXC 2180 ‘Image-Guided and Functionally Instructed Tumor Therapies’, University of Tuebingen, Tuebingen, Germany; ^3^ German Cancer Consortium (DKTK), partner site Tuebingen, a partnership between German Cancer Research Center (DKFZ) and University Hospital Tuebingen, Tuebingen, Germany; ^4^ School of Business and Economics, Faculty of Economics and Social Sciences, University of Tuebingen, Tuebingen, Germany; ^5^ Institute of Pathology and Neuropathology, University Hospital Tuebingen and Comprehensive Cancer Center, Tuebingen, Germany; ^6^ Institute of Tissue Medicine and Pathology (ITMP), University of Bern, Bern, Switzerland

**Keywords:** multiplexed tissue imaging, tumor microenvironment (TME), tumor immunophenotyping, single-cell analysis, spatial analysis workflow, MACSima™

## Abstract

Deciphering cellular components and the spatial interaction network of the tumor immune microenvironment (TIME) of solid tumors is pivotal for understanding biologically relevant cross-talks and, ultimately, advancing therapies. Multiplexed tissue imaging provides a powerful tool to elucidate spatial complexity in a holistic manner. We established and cross-validated a comprehensive immunophenotyping panel comprising over 121 markers for multiplexed tissue imaging using MACSima™ imaging cyclic staining (MICS) alongside an end-to-end analysis workflow. Applying this panel and workflow to primary cancer tissues, we characterized tumor heterogeneity, investigated potential therapeutical targets, conducted in-depth profiling of cell types and states, sub-phenotyped T cells within the TIME, and scrutinized cellular neighborhoods of diverse T cell subsets. Our findings highlight the advantage of spatial profiling, revealing immunosuppressive molecular signatures of tumor-associated myeloid cells interacting with neighboring exhausted, PD1^high^ T cells in the TIME of hepatocellular carcinoma (HCC). This study establishes a robust framework for spatial exploration of TIMEs in solid tumors and underscores the potency of multiplexed tissue imaging and ultra-deep cell phenotyping in unraveling clinically relevant tumor components.

## Introduction

Gaining a deeper understanding of protein expression, cellular compositions, and cell-cell interactions is key to understanding molecular principles in health and disease. Single-cell analyses have significantly enriched our understanding of cellular signatures and dynamics in cancer, analyzing tumor heterogeneity, immune cell infiltration, and immunotherapy-related patient outcomes (1–3). However, spatial investigations using single-cell sequencing approaches are limited due to disrupting tissue structures by homogenization. In contrast, tissue architectures can be explored and pathologically characterized by immunohistochemistry (IHC) and immunofluorescence (IF), both based on a finite set of analyzed markers. While a small set of markers can answer singular questions, a complex tissue composition like the tumor immune microenvironment (TIME) can only be deciphered with more information, meaning more markers.

To overcome this hurdle, IHC and IF were complemented by multiplexed imaging approaches (4–24). Multiplexed imaging techniques remarkably advanced spatial investigations and rely on optical, cytometric, or mass-spectrometry-based readouts. These applications are applied in 20-60-plex experiments by working with iterative cycles or multiparametric combinations of antibodies, labeled with fluorochromes, DNA barcodes, or metal tags. However, multiplexed imaging methods have limitations as well, such as the preservation of sample integrity or spatial resolution. Additionally, most multiplexed imaging approaches are restricted to a certain number (up to 60) of antibodies, due to specific antibody requirements or methodological limitations. Ultimately, the design and validation of new antibody panels can be laborious, complex, and costly, which makes pre-validated panels very useful (25).

We used the MACSima™ imaging cyclic staining (MICS) multiplexed imaging technology to overcome the hurdles of classical IF and other multiplexed imaging techniques. For this study, we generated and validated an antibody panel of more than 121 markers allowing for the precise description of immune cells within the TIME of heterogeneous carcinoma samples. In previous studies, multiplexed tissue analyses were used to characterize different cell subsets in cancerous tissue (26, 27). These investigations are focused on either a thorough description of the tumor or the associated immune cells. When tumor and TIME were described together in previous studies, the analysis depth was constrained by a limited number of markers (28, 29) or the imaging data sets were expanded by multi-omics approaches (30, 31). Yet, being able to stain and characterize tumor, stromal, and immune cells within the TIME together in greater depth by MICS highly multiplexed imaging is a new way of understanding cell compositions and drawing conclusions about the cellular interactome in solid tumors.

Particularly in clinical biomarker discovery, comprehensive spatial multi-omics approaches will gain novel insights into risk scarification and response prediction, e.g. demonstrated for anti-PD1 immune checkpoint blockade (ICB) in cutaneous T cell lymphoma (25, 32). ICB has dramatically changed the therapeutic landscape for multiple cancer entities. In advanced melanoma, combinatorial ICB with anti-CTLA4 plus anti-PD1 or anti-PD1 plus anti-LAG3 resulted in median progression-free survival of 11.5 (33) and 10.1 (34) months, respectively. In contrast, response rates to ICB across all cancer patients, irrespective of cancer type, are anticipated to be below 12.5% (35). Clearly, there is a huge clinical need for rational, biomarker-driven therapy decisions, evaluation and elucidation of therapy failure, and identification of combinatorial strategies (36). It is of utmost importance to investigate the expression landscape of immune-modulating proteins and unravel the complex cellular interaction neighborhoods at a spatial single-cell resolution. Therefore, we designed our panel to describe cellular phenotypes in a holistic manner and in their spatial context within the TIME. To demonstrate general feasibility, we analyzed a broad spectrum of cancer entities, focusing on colorectal cancer (CRC), prostate carcinoma (PCa), and two types of liver cancer: Intrahepatic cholangiocellular carcinoma (CCC) and hepatocellular carcinoma (HCC), representing leading causes of death worldwide (37–39). We describe distinct phenotypic architectures, specific tumor markers of CRCs, PCas, CCCs, and HCCs, and ultra-deep phenotyping of the TIME. This study demonstrates comprehensive multiplexed tissue immunophenotyping, profiling of cellular states, and functional spatial neighborhood analysis in unprecedented depth and complexity. Our study provides researchers with a validated versatile immuno-oncology antibody panel, combined with an easy-to-use analysis workflow. Panel and workflow have been utilized for and can be applied to various cancer entities and immune cell subsets, demonstrating their value as a powerful resource for addressing a multitude of immuno-oncology-related research questions.

## Materials and methods

### Patients and samples

Patient material was obtained via the biobank of the Institute of Pathology and Neuropathology of the University Hospital Tübingen. Respective tissue sections were considered irrelevant for diagnostic purposes by the responsible pathologist. All patient material included in this study was retrieved from the central biobank of the Comprehensive Cancer Centre Tübingen. All patients gave their written informed consent for biobanking and the use of biomaterials and clinical data for scientific assessment, as approved by the ethics committee of the University Hospital Tübingen (ethics approval No. 508-2016BO1) in accordance with the Declaration of Helsinki. To test our panel on diverse tumor types showing distinct architectures and representing major cancer types, we decided to include CRC, PCa, and different types of liver cancer, namely CCC and HCC. Patient characteristics are listed in **Supplementary Table S2**. Tissue identity was validated by a board-certified pathologist. Antibody validation was performed on healthy tonsil tissue, received from the Department of General, Visceral, and Transplant Surgery at the University Hospital Tübingen. Fresh or frozen tissue was embedded in Tissue-Tek^®^ O.C.T.™ Compound (Sakura Finetek USA) in cryomolds (25375-500, Polysciences, Inc.). Slow and controlled freezing was performed in an aluminum dish containing viscous ethanol, altogether cooled in liquid nitrogen. Embedded samples were stored at -80°C and equilibrated to the cutting temperature in the cryostat chamber for at least 20 mins before sectioning. Sections of 4-5 µm were cut with a cryostat (Leica CM 1950, Leica Biosystems) and placed onto SuperFrost^®^ plus slides (R. Langenbrinck GmbH). Slides were stored until the experimental procedure at -80°C.

### Tissue preparation for MICS

Frozen slides were incubated with 4% paraformaldehyde solution (J19943.K2, Thermo Scientific) for 10 mins at room temperature, washed three times with MACSima™ Running Buffer (130-121-565, Miltenyi Biotec), and immediately mounted onto an appropriate MACSwell™ Imaging Frame (One: 130-124-673, Two: 130-124-675, or Four: 130-124-676, Miltenyi Biotec). Sections were covered with MACSima™ Running Buffer until initial 4′,6-diamidino-2-phenylindole (DAPI) staining. Primary DAPI staining was performed right before the experiment by removing the MACSima™ Running Buffer and adding a 1:10 DAPI (130-111-570, Miltenyi Biotec) dilution in Running Buffer. After 10 mins incubation at room temperature, the samples were washed three times with Running Buffer and covered with the final sample volume. Staining and washing volumes depend on the respective working volumes for the Imaging Frames (One:1000 µl, Two:500 µl, Four: 250). MACSwell™ Imaging Frames were sealed with MACSwell™ Sealing Foil (130-126-866, Miltenyi Biotec) to prevent evaporation and protect the samples from contaminants.

### Antibodies and reagents for MICS

Primary fluorochrome-labeled antibodies conjugated to fluorescein isothiocyanate (FITC) or phycoerythrin (PE) were used for MICS. All antibody clones, dilutions, and fluorochrome recommendations are documented as our immune-oncology panel in **Table 1**. Additionally, we recommend 34 alternative fluorochromes which we tested on diverse tissues and which could be used for precise individual panel design, adding up to a total of 155 validated antibody conjugates (**Table 1**). Moreover, we provide antibodies that did not fulfill our validation criteria (**Supplementary Table S1**). Antibodies were prepared in a MACSwell™ Deepwell Plate (130-126-865, Miltenyi Biotec) in the appropriate dilution with MACSima™ Running Buffer for the respective MACSwell™ imaging frame working volumes. Before pipetting the antibodies into the Deepwell Plate, we recommend centrifuging the tubes for 20 sec at 1000 x g to sediment possible precipitates. DAPI was added to the antibody-containing Deepwell Plate in a 1:50 dilution in every eighth antibody cycle. The antibody-containing Deepwell Plates were sealed with MACSwell™ Sealing Foil (130-126-866, Miltenyi Biotec) to prevent evaporation.

**Table 1:** Immunophenotyping panel for multiplexed tissue imaging of cancer.

Antigen	Clone	Dilution	Order No.	Fluorochrome (+ tested alternatives)	Company
Actin (smooth muscle)	REAL650	50	130-123-363	FITC	Miltenyi Biotec
AFP	C3	50	sc-8399PE	PE	Santa Cruz Biotechnology
B7-H4	MIH43	100	358104	PE	BioLegend
Bcl-2	REA872	50	130-114-230	FITC	Miltenyi Biotec
Bcl-xL	H-5	200	Sc-8392PE	PE	Santa Cruz Biotechnology
CCL18	REA487	50	130-107-608	PE	Miltenyi Biotec
CD10	REAL318	50	130-118-368	FITC	Miltenyi Biotec
CD105	REA794	50	130-112-169	FITC	Miltenyi Biotec
CD107a	REA792	50	130-111-620	FITC	Miltenyi Biotec
CD112	REA1195	50	130-122-770	PE	Miltenyi Biotec
CD117	REA787	50	130-111-592	PE (APC)	Miltenyi Biotec
CD11a	REA378	50	130-124-886	FITC	Miltenyi Biotec
CD11b	REA713	50	130-110-552	FITC	Miltenyi Biotec
CD11c	REA618	50	130-113-587	PE	Miltenyi Biotec
CD123	REA918	50	130-115-263	FITC (APC)	Miltenyi Biotec
CD133	REA820	50	130-112-195	PE	Miltenyi Biotec
CD138	REA929	50	130-115-479	PE (APC)	Miltenyi Biotec
CD146	REA773	50	130-111-322	PE	Miltenyi Biotec
CD15	VIMC6	50	130-113-484	FITC	Miltenyi Biotec
CD155	REA1081	50	130-118-998	PE	Miltenyi Biotec
CD163	REA812	50	130-112-132	FITC	Miltenyi Biotec
CD169	REA1176	50	130-121-106	PE	Miltenyi Biotec
CD171	L1-OV198.5	200	371604	PE	BioLegend
CD183	REA232	50	130-120-452	PE	Miltenyi Biotec
CD184	REA649	50	130-117-504	PE	Miltenyi Biotec
CD19	LT19	50	130-113-168	FITC	Miltenyi Biotec
CD196	REA190	50	130-120-458	PE	Miltenyi Biotec
CD1c	AD5-8E7	50	130-113-301	FITC	Miltenyi Biotec
CD2	REA1130	50	130-119-508	PE	Miltenyi Biotec
CD20	REA1087	50	130-118-292	FITC (PE)	Miltenyi Biotec
CD200	REA1067	50	130-118-128	FITC	Miltenyi Biotec
CD206	DCN228	50	130-123-671	FITC (PE)	Miltenyi Biotec
CD21	REA940	50	130-115-609	FITC	Miltenyi Biotec
CD22	REA340	50	130-124-223	FITC	Miltenyi Biotec
CD24	REA832	50	130-112-656	PE	Miltenyi Biotec
CD243	REA495	50	130-124-440	PE (APC)	Miltenyi Biotec
CD25	REA945	50	130-115-534	PE (APC)	Miltenyi Biotec
CD25	M-A251	50	356104	PE	BioLegend
CD26	FR10-11G9	50	130-126-362	PE	Miltenyi Biotec
CD27	REA499	50	130-113-639	FITC (PE)	Miltenyi Biotec
CD276	REA1094	50	130-118-570	PE (FITC)	Miltenyi Biotec
CD3	REA1151	50	130-120-267	FITC (APC)	Miltenyi Biotec
CD31	REA1028	50	130-117-224	FITC	Miltenyi Biotec
CD314	REA1228	50	130-124-341	PE	Miltenyi Biotec
CD34	REA1164	50	130-120-515	PE	Miltenyi Biotec
CD36	REA760	50	130-110-739	FITC	Miltenyi Biotec
CD38	REA671	50	130-117-717	PE (APC)	Miltenyi Biotec
CD39	REA739	50	130-110-650	PE	Miltenyi Biotec
CD4	REA623	50	130-114-531	FITC (PE)	Miltenyi Biotec
CD40	REA733	50	130-110-946	PE	Miltenyi Biotec
CD44	REA690	50	130-113-342	PE (FITC)	Miltenyi Biotec
CD45	REA747	50	130-110-632	PE	Miltenyi Biotec
CD45RA	T6D11	50	130-113-355	FITC	Miltenyi Biotec
CD45RO	UCHL1	50	130-113-549	FITC	Miltenyi Biotec
CD47	REA220	50	130-123-754	PE	Miltenyi Biotec
CD48	REA426	50	130-106-516	PE	Miltenyi Biotec
CD49α	REA1106	11	130-119-305	FITC	Miltenyi Biotec
CD49β	REA188	11	130-100-337	FITC	Miltenyi Biotec
CD54	REA266	50	130-120-711	PE	Miltenyi Biotec
CD56	AF12-7H3	50	130-113-307	PE (APC)	Miltenyi Biotec
CD57	REA769	50	130-111-810	PE	Miltenyi Biotec
CD61	REA761	50	130-110-748	FITC	Miltenyi Biotec
CD64	REA987	50	130-116-195	FITC	Miltenyi Biotec
CD66b	REA306	50	130-123-694	FITC	Miltenyi Biotec
CD69	FN50	50	130-113-524	FITC (PE)	Miltenyi Biotec
CD70	REA292	100	130-104-307	FITC	Miltenyi Biotec
CD71	REA902	50	130-115-028	FITC (PE)	Miltenyi Biotec
CD73	REA804	50	130-111-908	PE (APC)	Miltenyi Biotec
CD74	REA1103	50	130-119-026	PE	Miltenyi Biotec
CD8	REA734	50	130-110-677	FITC	Miltenyi Biotec
CD9	REA1071	50	130-118-806	FITC	Miltenyi Biotec
CD90	REA897	50	130-114-859	FITC	Miltenyi Biotec
CD95	REA738	50	130-113-004	PE (FITC)	Miltenyi Biotec
CD96	REA195	100	130-101-032	PE	Miltenyi Biotec
CD99	REA1174	50	130-121-078	PE	Miltenyi Biotec
Collagen III	REAL912	50	130-127-357	PE	Miltenyi Biotec
Collagen IV	REAL567	50	130-122-866	PE	Miltenyi Biotec
CSF1R	12-3A3-1B10	100	NBP1-43362PE	PE	Novus Biologicals
CTLA-4	BNI3	200	369604	PE	BioLegend
Desmin	REA1134	50	130-119-489	FITC (APC, PE)	Miltenyi Biotec
EpCAM	REA764	50	130-110-998	FITC	Miltenyi Biotec
Fibronectin	REAL555	50	130-122-864	PE	Miltenyi Biotec
FOLR2	NBP2-99741	100	NBP2-99741F	FITC	Novus Biologicals
FoxP3	236A/E7	50	12-4777-42	PE	Thermo Fisher Scientific
FSP-1	NBP2-36431	200	NBP2-36431F	FITC	Novus Biologicals
Galectin 9	REA435	50	130-124-237	PE (APC)	Miltenyi Biotec
GD2	14.G2a	50	562100	PE (FITC)	BD
GFAP	REA335	50	130-118-351	PE	Miltenyi Biotec
Glypican 3	307801	200	FAB2119G	FITC	RnD Systems
H2AX	REA502	50	130-125-883	PE	Miltenyi Biotec
HER2	REA1232	50	130-124-466	PE	Miltenyi Biotec
HIF-1	EP1215Y	200	ab190197	FITC	Abcam
HLA-ABC	REA230	50	130-120-055	PE (APC, FITC)	Miltenyi Biotec
HLA-DR	REA805	50	130-111-789	PE (APC, FITC)	Miltenyi Biotec
HLA-DR/DP/DQ	REA332	50	130-120-715	PE	Miltenyi Biotec
HNF-4α	H-1	50	sc-374229PE	PE	Santa Cruz Biotechnology
Hsp70	REA349	50	130-124-694	FITC	Miltenyi Biotec
IDO	D5J4E	100	103125	PE	Cell Signaling Technology
Ki-67	REA183	50	130-117-691	FITC	Miltenyi Biotec
LAG3	REA351	25	130-120-470	PE	Miltenyi Biotec
MUC1	16A	50	355604	PE	BioLegend
Myosin	REA1107	100	130-119-313	FITC	Miltenyi Biotec
NANOG	REA314	50	130-117-377	PE	Miltenyi Biotec
p16	F-12	50	sc-1661PE	PE	Santa Cruz Biotechnology
p21	F-8	50	sc-271610PE	PE	Santa Cruz Biotechnology
p53	REA1132	50	130-119-502	PE	Miltenyi Biotec
Pan-Cytokeratin	REA831	50	130-112-743	FITC (APC, PE)	Miltenyi Biotec
PD-1	REA1165	50	130-120-382	PE	Miltenyi Biotec
PDGFR β	MAB1263	200	FAB1263T-100UG	FITC	RnD Systems
PD-L1 1	REA1197	50	130-122-809	PE	Miltenyi Biotec
PD-L1 2	MIH2	50	393608	PE	BioLegend
Podoplanin	REAL468	50	130-125-009	FITC	Miltenyi Biotec
PSMA	GCP-04	100	NBP1-45057AF488	FITC	Novus Biologicals
S100A8	REA917	50	130-115-253	FITC (APC, PE)	Miltenyi Biotec
S100A9	REA859	50	130-114-515	FITC	Miltenyi Biotec
SSEA-1 (CD15)	REA321	50	130-117-689	PE	Miltenyi Biotec
TIM3	REAL818	50	130-125-682	PE	Miltenyi Biotec
Vimentin	REA409	50	130-123-774	PE (APC, FITC)	Miltenyi Biotec
VISTA	D1L2G	200	18946	PE	Cell Signaling Technology
β-Actin	REA1148	50	130-120-276	FITC	Miltenyi Biotec
β-Catenin	REA480	50	130-123-546	FITC (APC, PE)	Miltenyi Biotec

### MICS

The MACSima™ Imaging System provides a fully automated workflow for iterative cycles of immunofluorescence sample staining, multi-field imaging, and fluorochrome removal (photobleaching or enzymatic digest). The sample slides mounted with a MACSwell ™ Imaging Frame as well as the antibody-containing MACSwell™ Deepwell Plate were placed on the xy-stage which moves reagents and samples to the correct needle and microscope positions. Antibody incubation was performed for 10 mins (standard setting), followed by excitation of the fluorochromes by specific laser/filter combinations and detection with a monochromatic scientific CMOS camera. A detailed description of the MACSima™ hardware (liquid handling system, microscope, stage) was published previously (40). We used hardware version 1.5.0 as well as software version 0.13.2 for image acquisition.

Before the iterative cycles were initiated, ROIs were defined and the focus was adjusted. ROIs were defined based on the DAPI signal shown in the overview scan and are based on customized numbers of overlapping fields of view (FoV). Additionally, we performed an H&E staining of a sequential tissue section and used this H&E stain as a reference which ensured optimal region definition. The focus was primarily set using the hardware autofocus option and adjusted manually for each ROI based on the DAPI signal. The MACSima™ captures various raw images of each FoV and each cycle. These images are utilized for subsequent image processing performed in MACS iQ View. One imaging cycle includes the following: DAPI image (used for FoV stitching), as well as bleach and staining images for each channel captured at three different exposure times (used for background subtraction and optimal exposure selection). After the MICS experiment, slides were H&E stained according to pathological standards (41) and imaged with a slide scanner (Panoramic Midi II, 3D Histech).

### Image processing

Individual raw FoV images were processed with the automated pipeline on the MACS iQ View software (Version 1.2.2). Detailed information on image processing was described before (40). In brief, different exposure times are combined in a high dynamic range image, normalizing based on multiple exposures when overexposure is detected. Exposure selection was set for “automatic”, validated and, if necessary, adjusted to optimal exposure times. Flatfield and distortion corrections are applied for the calibration data of the individual instrument. Next, neighboring FoV are automatically stitched together based on the DAPI staining of overlapping margins. Finally, the pre-stain bleach images of each cycle, acquired before the subsequent antibody incubation and showing potential residual staining of the previous cycle, are subtracted from the subsequent staining image, resulting in the final processed and stitched images. The images were cropped to DAPI based on experimental values of 200 pixels for top/bottom and 100 pixels for right/left correction.

### Analysis workflow using MACS iQ View

Image datasets for each ROI, including all staining and autofluorescence images in TIFF format, were integrated into the MACS iQ View software (Version 1.2.2). Advanced cell segmentation was performed using the MACS iQ View segmentation pipeline. In detail, segmentation is conducted based on nuclear and cytoplasmic markers. We used the “Advanced Morphology for Tissue” nuclear detection option based on the first DAPI staining. Min/Max Diameter, Detection Sensitivity, Separation Force, and Smoothing Filter Sigma were adjusted based on the DAPI staining intensity and the respective tissue, see **Supplementary Table S3**. Cytoplasmic signal was allocated to single cells by using the “Constrained Donut” option based on automatically chosen constraint channels. Detection Sensitivity and Donut Width were specified for each ROI individually based on signal intensities (**Supplementary Table S3**). We provide recommended parameters for efficient cell segmentation for densely packed and hard-to-segment tissues, like tonsils, and adapted settings for various cancerous tissues based on tumor morphology. Depending on staining efficiency, exposure time, and tissue composition the recommended parameters need to be adjusted. Therefore, we give guidelines for the optimal usage of segmentation parameters. Segmentation was cross-validated via the visual control in MACS iQ View by displaying nuclear and cytoplasmic markers overlayed by the segmentation mask.

Antibody cross-validation was performed for each antibody, dilution, and experiment. Having the ability to stain several markers for a specific cell type, we compared markers of a single experiment (in-run comparison) and between different experiments. Additionally, we included a reference tissue (tonsil) on each slide for the in-run staining control. As a third reference, we used public databases like the Human Protein Atlas (proteinatlas.org (42)) or classical IHC staining to compare the MACSima™ staining and validate the antibodies.

Marker expression was allocated to the segmented cells and was used for expert-based gating of cell populations and cell type annotation (exemplary gating strategy: **Supplementary Figure S2A**). Additional markers that were not used for gating were displayed as Color Maps to ascertain the cell type annotation (**Supplementary Figure S2B**). Gates were directly side-by-side compared with the staining image in the MACS iQ View image control window. Expert-based (sub-)gating of cell populations was adjusted for ROI and cell type-specific cell expression patterns, depicted as Scatter Plots and Histograms in MACS iQ View. Using the “Merge” operator, gated cell populations from different sub-gates were integrated into a combined cell type gate.

Neighborhood analysis of annotated cell types (i.e. PD1^low/high^ T cells) was conducted with the MACS iQ View distance analysis tool, applied for an annotated cell type of interest. Based on the distance histogram, we defined a range of 5 µm (≙ 47,14 pixels) or 25 µm (≙ 235,85 pixels) around the annotated cell type of interest. All cells within the distance range were merged (“Merge” operator, option “AND”) with all pre-defined annotated cell types. Cells present in both an annotated cell type and the range around the cell type of interest resulted in a new intersection population.

An exemplary MACS iQ View workflow script containing advanced segmentation settings, expert-based gating of cell populations, cell type annotation, and distance analysis is deposited at Zenodo: 10.5281/zenodo.10057717.

### Bioinformatical analyses

After advanced cell segmentation using the MACS iQ View, data was exported as a CSV file and analyzed with R (version 4.1.0). To limit the effect of potentially spurious outliers, we applied winsorization to the raw expression data, capping values at the 1st and 99th percentiles. We computed percentiles separately for every marker. Next, to reveal patterns in the data that are not apparent in the original, antibody-dependent scale, we applied the inverse hyperbolic sine transformation (arcsinh transformation) to the winsorized data. Replicable code for the analyses is available on Zenodo: 10.5281/zenodo.10057717.

### Data visualization

Graphs were produced using GraphPad Prism (Version 9.5.1) and images were exported from MACS iQ View (Version 1.2.2). We created the faceted violin plots using the {ggplot2}-library (43).

## Results

### Establishment and validation of a comprehensive MICS panel and analysis workflow

**Figure 1:**
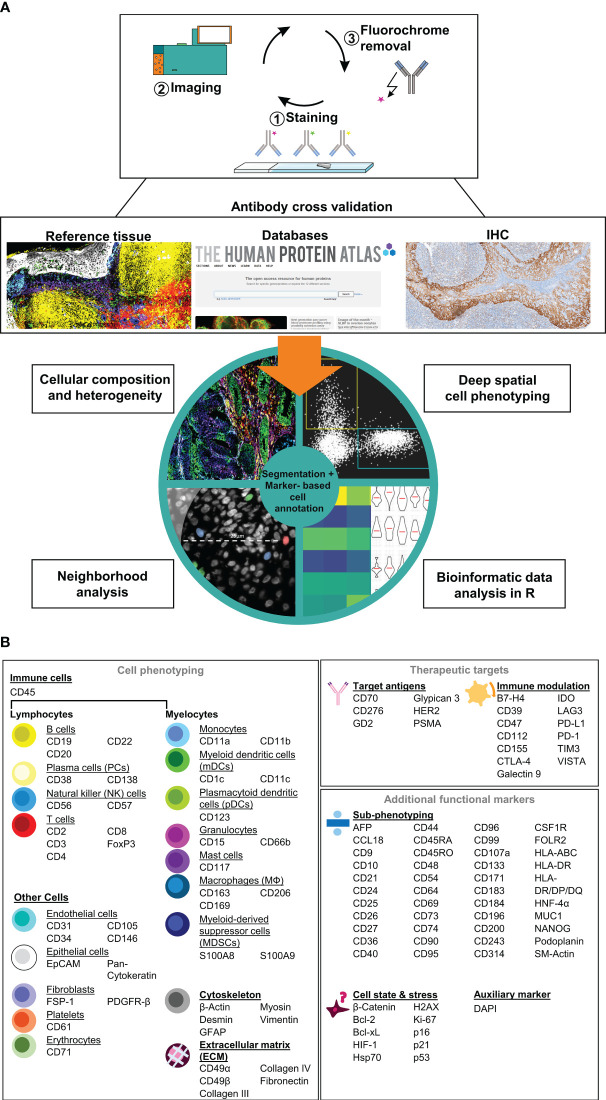
Immunophenotyping panel and analysis workflow for MACSima™ imaging cyclic staining (MICS). **(A)** In a cyclic fashion, MICS is based on staining a specimen with fluorochrome-labeled antibodies, epifluorescence imaging, and removal of the fluorochrome. Resulting antibody staining were cross-validated using reference tissue, databases, or classical immunohistochemistry (IHC) staining and were subsequently used for a marker-based cell annotation. Based on advanced cell segmentation, cell types can be annotated, cellular composition and heterogeneity analyses as well as deep profiling and neighborhood analyses can be performed. CSV files can be exported for external bioinformatic analyses. **(B)** Validated antibody panel including 121 antibodies for 118 targets for cell (sub-)phenotyping of immune and tumor cells as well as for identification of therapeutical targets.

Here, we describe the establishment, validation, and application of a comprehensive immunophenotyping panel for MICS, tailored to address immuno-oncology research questions. MICS multiplexed tissue imaging is based on iterative cycles of 1) fluorochrome-labelled antibody staining, 2) epifluorescence imaging, and 3) fluorochrome removal (**Figure 1A**, *top*). After image processing, staining accuracy was assessed through cross-validation by co-staining of reference tissues as well as comparing staining patterns with publicly accessible databases and traditional IHC staining (**Figure 1A**, *middle*). Following antibody cross-validation, we performed advanced segmentation analysis within the MACS iQ View software and used the ascertained marker expression profiles for expert cell annotation, which serves as a basis for 1) defining cellular composition, heterogeneity, and spatial distribution, 2) performing deep spatial cell phenotyping, 3) analyzing cellular neighborhoods, and 4) utilizing data for external bioinformatic analysis pipelines (**Figure 1A**, *bottom*). In detail, image processing, image quality control, antibody cross-validation, advanced segmentation, marker-based cell annotation, and data analysis of all markers were performed within the MACS iQ View software. For optimal advanced cell segmentation and applicability for various tissues, we provide recommended parameters and guidelines (**Supplementary Table S3**). Utilizing the advantages of the MACS iQ View software, the expert-based gating analysis (exemplary gating strategy in **Supplementary Figure S2A**) was side-by-side controlled with the staining image and scatter plot gating was complemented by depicting additional marker expressions as Color Maps (**Supplementary Figure S2B**). For external downstream analyses, segmentation-based expression data as well as annotated cell subtypes were further analyzed in R, thereby combining the strengths of MACS iQ View and R in our analysis workflow. In the current study, we present our immunophenotyping panel comprising 121 antibodies addressing 118 antigen markers (**Figure 1B**). Detailed information on clones, vendors, tested fluorochromes, and suggested dilution is provided in **Table 1**. In addition to the core panel used in this study, we tested alternative fluorochrome conjugates across various tissues, resulting in a total of 155 validated antibody conjugates for multiplexed imaging (**Table 1**). We provide optional fluorochrome recommendations to facilitate further customization and optimization of our immunophenotyping panel. Essentially, we emphasized having more than two markers to distinguish distinct cell types, functioning as an internal staining control and strengthening the analytical power. The immunophenotyping panel is suitable for cell phenotyping of at least 16 different cell populations (lymphocytes: B cells, plasma cells (PCs), natural killer (NK) cells, T cells; myelocytes: Monocytes, myeloid dendritic cells (mDCs), plasmacytoid dendritic cells (pDCs), granulocytes, mast cells, myeloid-derived suppressor cells (MDSCs), macrophages (MΦ); endothelial cells, epithelial cells, fibroblasts, platelets, erythrocytes), as well as for definition of the cellular cytoskeleton and extracellular matrix (ECM) components. To further dissect the cellular landscape, we included 40 markers for ultra-deep sub-phenotyping, resulting in highly defined and diversified cell populations and cellular states including markers for characterizing cellular stress and senescence. Moreover, we integrated markers for possible therapeutic intervention, especially for immunotherapeutic targeting with monoclonal antibodies (mAbs), chimeric antigen receptor (CAR) T cells, or ICB. Representative staining of all markers on tonsil tissue is provided in **Supplementary Figure S1**. Since panel design is laborious and costly, we additionally provide a list of tested antibodies that did not fulfill our validation criteria (**Supplementary Table S1**). Taken together, we provide a validated immunophenotyping panel plus an analytic workflow for multiplexed tissue imaging using MICS.

### Characterization of human tonsil tissue with a comprehensive immunophenotyping panel

**Figure 2:**
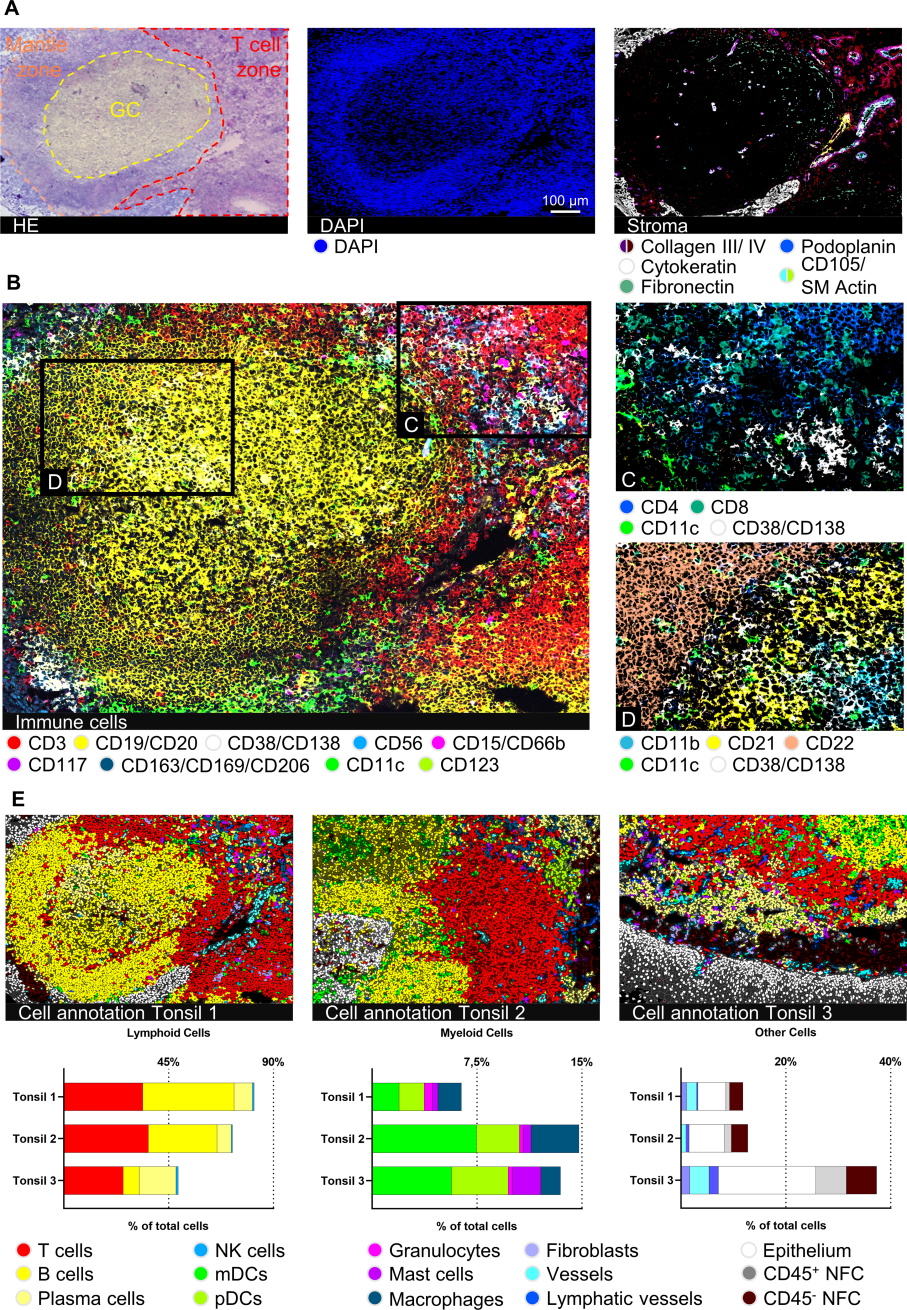
Deep spatial profiling of human palatine tonsil tissues. **(A)** Hematoxylin and eosin (H&E) staining after 92 MICS cycles, including the marked epithelium, germinal center (GC), and T cell zone of the lymphoid follicle. MICS DAPI and stroma staining depicting the composition and structure of the tonsil. Markers: Collagen III, collagen IV, fibronectin (all extracellular matrix (ECM), cytokeratin (epithelium), podoplanin (lymphatic vessels), CD105/SM Actin (blood vessels). **(B)** Immune cell content of a human palatine tonsil comprising T cells (CD3), B cells (CD19/CD20), plasma cells (PCs) (CD38/CD138), NK cells (CD56), granulocytes (CD15/CD66b), mast cells (CD117), macrophages (MΦ) (CD163/CD169/CD206), myeloid dendritic cells (mDCs) (CD11c), and plasmacytoid dendritic cells (pDCs) (CD123). **(C)** Detailed view on the T cell zone, mainly composed of CD4^+^ helper T cells (T_h_) and CD8^+^ cytotoxic T cells (T_c_), mDCs (CD11c), and PCs (CD38/CD138). **(D)** Detailed view on the GC-mantle zone border, showing different B cells (CD11b, CD21, CD22), mDCs (CD11c), and PCs (CD38/CD138). **(E)** Cell annotations of three different tonsil samples plus respective bar graphs of gated cell populations, comparing the cell content between the three tonsil samples. Depicted markers and annotated cell types as indicated by the color code. ROI sizes: 976 x 640 µm, zoomed-in subregions in **(C, D)**: 334 µm x 219 µm. Scale bar: 100 µm.

To validate our immunophenotyping panel and test the feasibility of the approach, we chose palatine tonsil tissue as a reference. Palatine tonsils are secondary lymphoid organs playing an important role in developing self-tolerance and establishing adaptive immunity (44). A plethora of cells of the innate as well as adaptive immune system in different activation or differentiation states can be found within tonsils, complemented by stromal and vasculature components (45). Therefore, tonsil tissue has been broadly used for antibody panel validation (26, 46, 47). Applying our panel to human tonsil sections enabled a highly detailed mapping of tonsillar immune cells (**Figure 2A**, *middle* and **Figure 2B**) and stromal components (**Figure 2A**, *right*). Despite performing 89-92 MICS cycles, including staining, imaging, and fluorochrome removal, which resulted in an experimental duration of more than seven days, the tissue integrity was preserved and used for final post-experimental H&E staining (**Figure 2A**). Advanced segmentation resulted in a total of 8074, 7379, and 6213 cells for tonsils 1, 2, and 3, respectively. We were able to classify 13 main cell types, namely T cells, B cells, PCs, NK cells, mDCs, pDCs, granulocytes, mast cells, MΦ, fibroblasts, blood, and lymphatic vessels, as well as squamous epithelial cells (**Figure 2C**). We further sub-characterized the T cells into CD4^+^ helper T cells (T_h_) and CD8^+^ cytotoxic T cells (T_c_) (**Figure 2C**). We could identify B cell subclusters in the germinal center (GC) organization, categorizing them into CD21^+^ germinal center B cells, CD22^+^ mantle zone B cells, and CD11b^+^ B cells (**Figure 2D**). Additional B cell activation states (CD25^+^, CD44^+^, CD69^+^) and memory phenotypes (CD40^+^) could be deciphered. In line with previous findings (48–52), we defined different architectural zones of the tonsil based on multiparametric staining (mantle zone: CD27^+^, CD39^+^; GC: CD9^+^, CD10^+^, CD171+; non-GC: BCL-2^+^, CD44^+^) (**Supplementary Figure S2C**). Expert marker-based gating using our analysis workflow allows definite cell annotation and quantification of immune cell subsets (**Figure 2E**), consistent with literature (53). Taken together, we validated an antibody panel of 121 markers using palatine tonsil tissue which serves as a platform to be adapted for a plethora of research questions.

### Immuno-oncological profiling of heterogeneous cancer tissues

**Figure 3:**
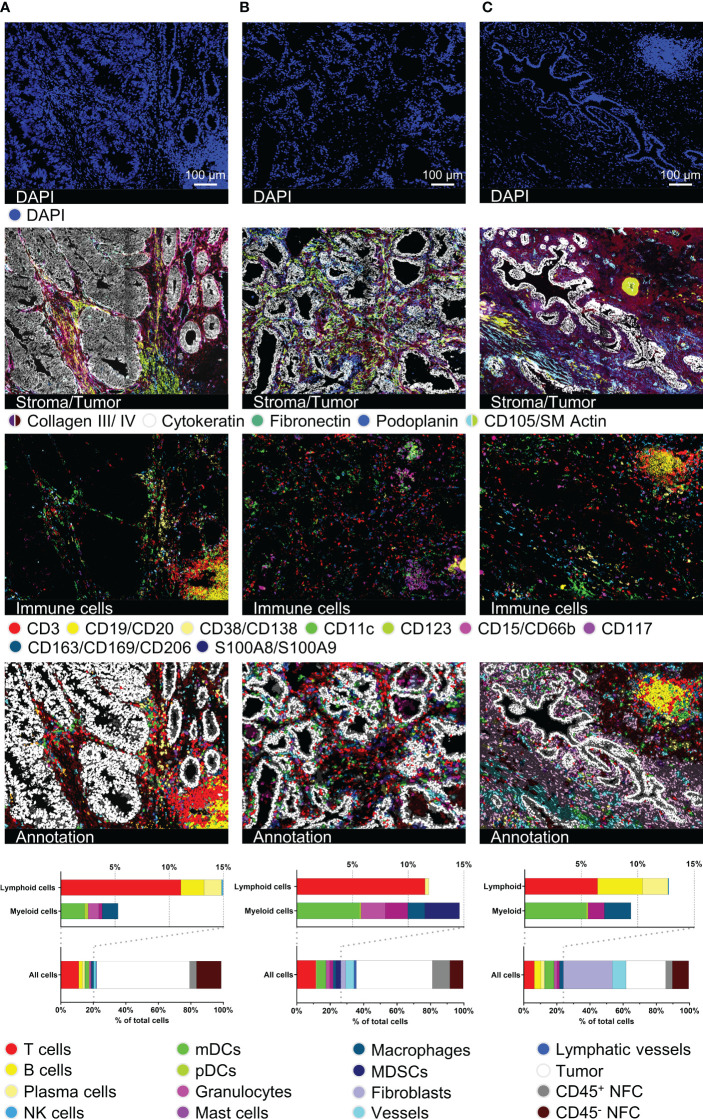
Immuno-oncological description and characterization of cancerous tissues. For all three tumor samples, DAPI, stroma- and tumor-characterizing images (collagen III, collagen IV, cytokeratin, fibronectin, podoplanin, CD105/SM Actin), immune cell content in a 15-color image, as well as cell type annotation images and bar graphs are shown. **(A)** represents a colorectal carcinoma (CRC), **(B)** a prostate carcinoma (PCa), and **(C)** an intrahepatic cholangiocellular carcinoma (CCC). Depicted markers and annotated cell types as indicated by the color code. ROI sizes: **(A)**: 975 x 747 µm, **(B)**: 974 x 747 µm, **(C)**: 974 x 769 µm. Scale bar: 100 µm.

Next, we validated our antibody panel on samples of three different cancer entities, particularly CRC, PCa, and CCC (**Figure 3**). The entities were chosen to provide a spectrum of histologically different tumors to demonstrate the broad applicability of the antibody panel. The diverse morphology as well as tissue integrity was preserved over 92 MICS cycles (H&E staining in **Supplementary Figure S3A**). Samples were cross-validated and analyzed using the presented analysis workflow (**Figure 1A**). Cytokeratin staining depicted the histological features of glandular CRC and PCa and ductal intrahepatic CCC, all originating from epithelial cells (**Figure 3**, *second row*). By using the markers for ECM and structural components, we differentiated the respective tumoral stroma differences of the three entities, consistent with literature (54–56). While we could define a reduced collagenous stroma for juxtaposed glands in CRC (cribriform, back-to-back aspect), (**Figure 3A**, *second row*), the PCa and intrahepatic CCC samples were dominated by a dense stroma consisting of collagen III and collagen IV and fibroblastic markers (**Figures 3B, C**, *second row*). Quantification of 36 different cancer-associated markers showed the heterogeneity of the different tumor types (**Supplementary Figure S3B**). We spatially mapped the expression of possible immunotherapeutic targets like EpCAM, uniformly expressed in all samples, and HER2, demonstrating a higher degree of intratumoral heterogeneity, particularly in the CRC sample. On the contrary, CD155 and CD243, e.g., were only detected in intrahepatic CCC, whereas PD-L1 was highly expressed in the CCC and CRC samples (**Supplementary Figures S3C–E**). Our data clearly demonstrates the feasibility of the applied technology to individually characterize tumor samples in terms of quantitative expression and spatial heterogeneity of druggable immune targets. This detailed tumor phenotyping can serve as a prerequisite for complex precision immunotherapy approaches.

In line with our findings in palatine tonsil tissue, we were able to identify different immune cell populations as well as tertiary lymphoid structures (TLS) within the tumor tissues (**Figure 3**, *third row*). Segmentation resulted in 7963 cells for the CRC, 5029 cells for the PCa, and 7101 cells for the CCC sample. Cells were annotated marker-based and we quantified the cellular components, including ten different immune cell types (T cells, B cells, PCs, NK cells, mDCs, pDCs, granulocytes, mast cells, MΦ, MDSCs), fibroblasts, vessels, lymphatic vessels, epithelial, and tumor cells. While percentages of total infiltrating immune cells (CRC: 20,24%, PCa: 26,45%, CCC: 23,91%) were similar, cell types and particularly spatial distribution differed dramatically, see **Figures 3A–C**. In both the CRC and the CCC samples, TLSs were identifiable (**Figures 3A, C**). In the CRC sample, the TLS presents as a peritumoral primary follicle-like structure with clearly distinguishable B and T cell zones and spotted mDCs. In contrast, the TLS in the CCC sample is located within a dense collagenous stroma and exhibits a more mature secondary follicle-like structure. GC reaction was defined by CD21^+^ GC B cells and surrounding CD34^+^/CD54^+^ high endothelial venules (HEV) (**Supplementary Figures S3F, G**, HEV highlighted by *white arrows*). In contrast, B cells were found to be absent in the PCa sample. Taken together, this set of data clearly validates the feasibility of our immunophenotyping panel to comprehensively dissect tissues from various cancer entities at a single-cell level to study functional cellular composition and spatial architecture. Additionally, it underscores the dramatic phenotypical differences between cancer entities and the eminent need for comprehensive spatial analyses to study and understand cancer biology and immunology.

### Detailed description of HCC intratumoral sub-regions

**Figure 4:**
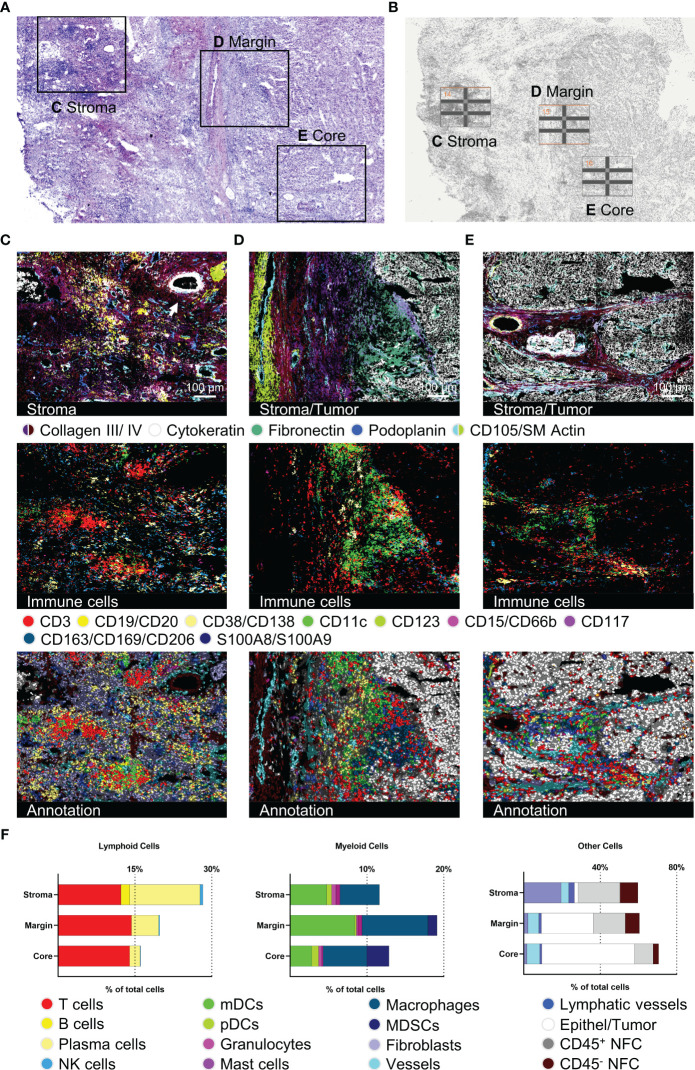
Detection of structural, immune, and tumor markers in three different hepatocellular carcinoma (HCC) tumor regions. **(A)** H&E staining after 92 MICS cycles plus marked regions of interest (ROIs). **(B)** Pre-run DAPI staining for ROI definition. **(C–E)** represent tumor-associated stroma area, tumor margin, and tumor core. For each ROI, images for stroma and tumor characterization (collagen III, collagen IV, cytokeratin, fibronectin, podoplanin, CD105/SM Actin), as well as immune cells (T cells, B cells, PCs, mDCs, pDCs, mast cells, MΦ, myeloid-derived suppressor cells (MDSCs)), and the cell type annotation are shown. **(F)** Quantifications of the annotated cell types by bar graphs are outlined for each ROI. Depicted markers and annotated cell types as indicated by the color code. ROI sizes: Tumor-associated stroma (ROI14): 975 x 770 µm, tumor margin (ROI15): 975 x 769 µm, tumor core (ROI16): 975 x 769 µm. Scale bar: 100 µm.

**Figure 5:**
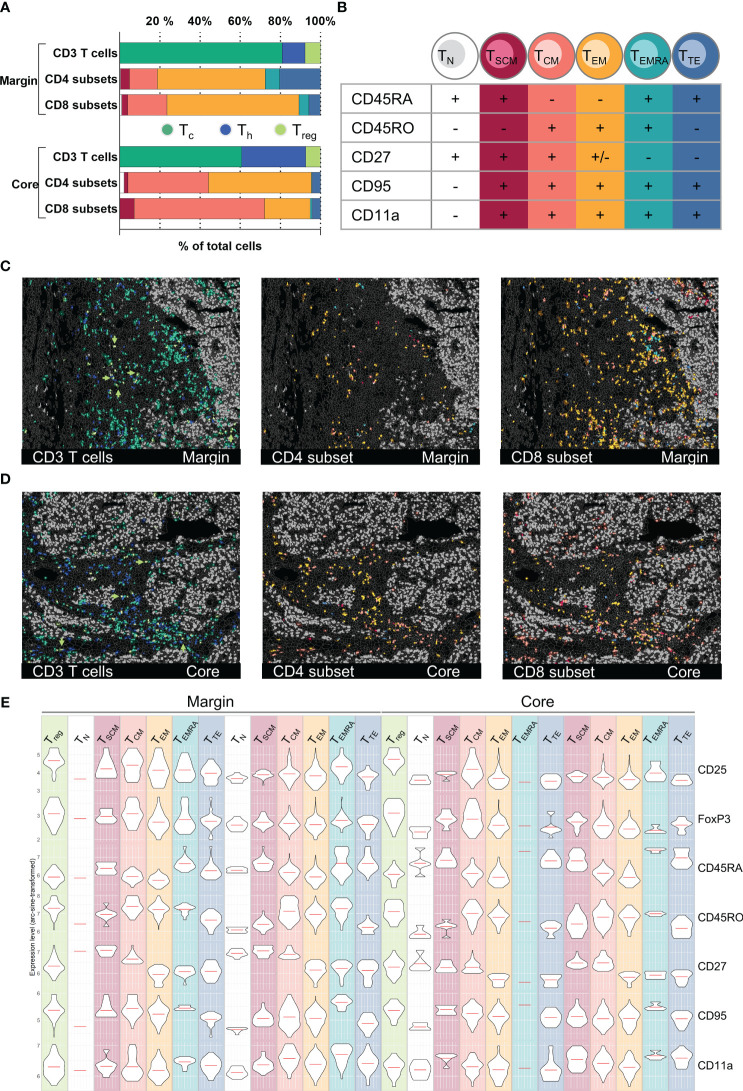
T cell subset classification and spatial distribution in an HCC sample. **(A)** Bar graph quantifications of gated T cell CD3^+^ T cells subpopulations (CD4^+^ T_h_, CD8^+^ T_c_, FoxP3^+^/CD25^+^ T_reg_) as well as T cell differentiation subsets (T_N_, T_SCM_, T_CM_, T_EM_, T_EMRA_, T_TE_) for HCC tumor margin and tumor core. **(B)** Subclassification criteria used for T cell differentiation subset gating. Detailed gating scheme see **Supplementary Figures S5A–D** Spatial distribution of CD3+ T cell subsets as well as CD4+ and CD8+ differentiation subsets for tumor margin **(C)** and tumor core **(D)**. Arrowheads in **(C, D)** highlight the spatial distribution of regulatory T cells (T_reg_). **(E)** Violin plots demonstrating expression levels of markers used for the definition of T cell differentiation subsets in tumor margin and tumor core: CD25, FoxP3, CD45RA, CD45RO, CD27, CD95, CD11a. Depicted markers and annotated cell types as indicated by the color code. ROI sizes: Tumor margin (ROI15) and tumor core (ROI16): 975 x 769 µm.

**Figure 6:**
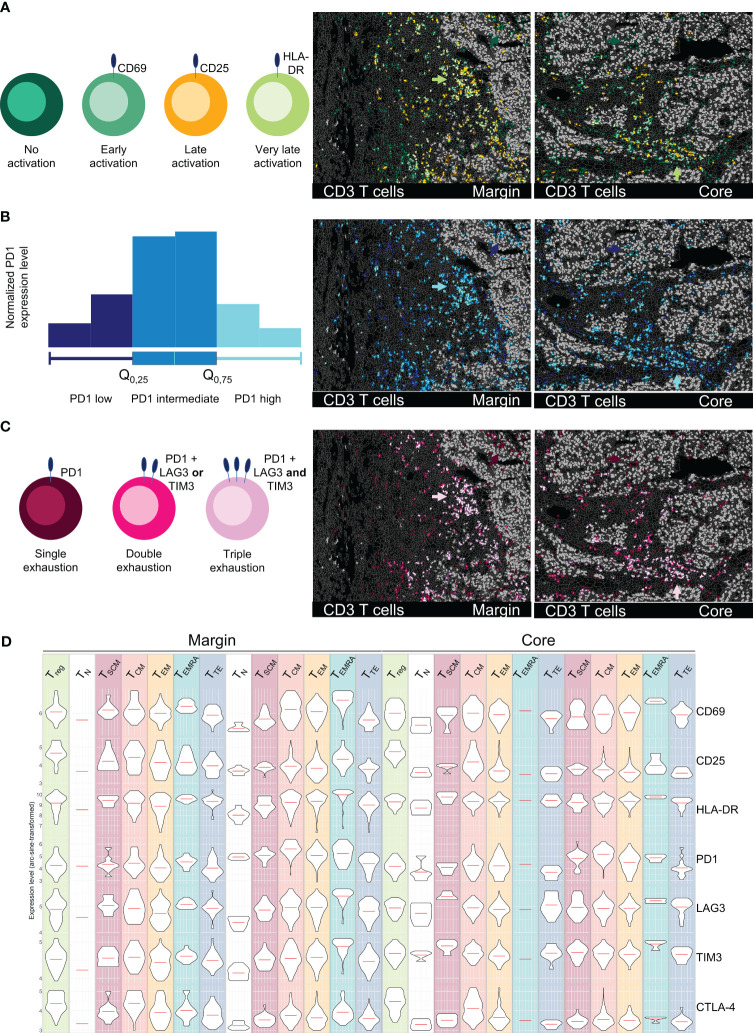
Spatial profiling of T cell activation and exhaustion states in an HCC sample. All images depict tumor margin (left) and tumor core (right). **(A)** Activation status of T cells subsets (CD69 = early, CD25 = late, HLA-DR = very late). **(B)** PD1 marker expression on T cells, categorized as low (0-25%), intermediate (25-75%), and high (75-100%). **(C)** Exhaustion levels of T cells based on the expression of PD1 (single exhaustion), PD1 plus TIM3 or LAG3 (double exhaustion), or PD1 plus TIM3 and LAG3 (triple exhaustion). Arrowheads in **(A–C)** highlight the ITM-proximal immune cell cluster in tumor margin and core. **(D)** Representation of activation/exhaustion markers for T cells (CD69, CD25, HLA-DR, PD1, LAG3, TIM3, CTLA4) as violin plots for T cell activation/exhaustion subsets. Depicted markers and annotated cell types as indicated by the color code. ROI sizes: Tumor margin (ROI15) and tumor core (ROI16): 975 x 769 µm.

**Figure 7:**
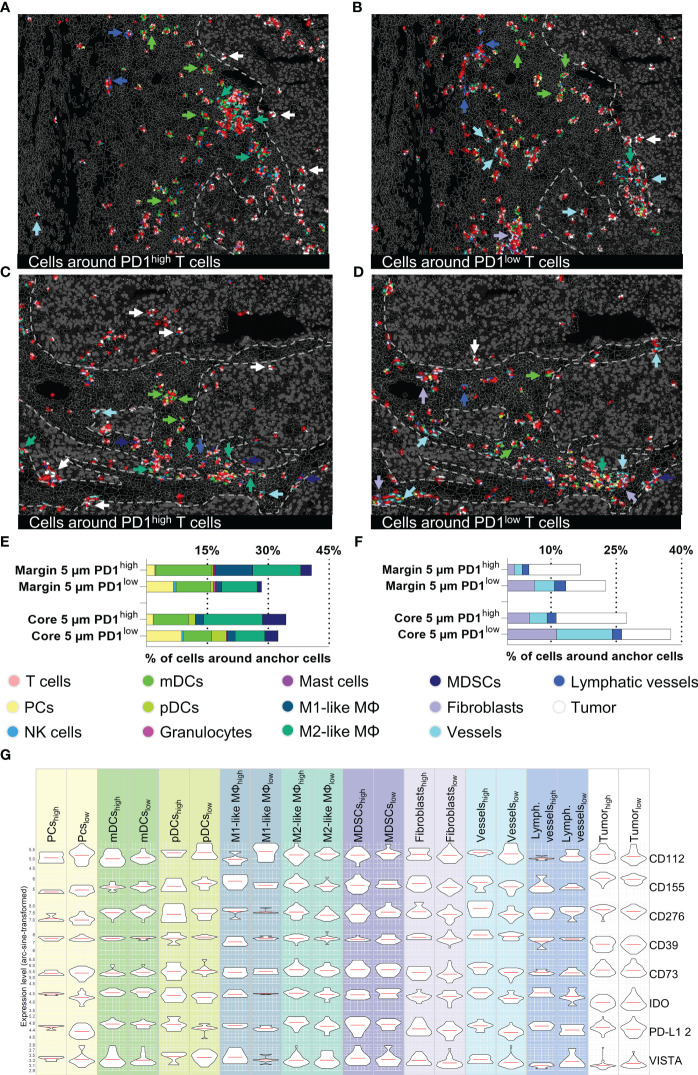
Cellular neighborhood analysis of PD1^high/low^ T cells in the tumor margin and core. **(A–D)** Topology of PD1^high^ (left) and PD1^low^ (right) T cells and their cellular neighborhood within a 5 µm range. **(A, B)** represent tumor margin and **(C, D)** show tumor core areas. Cell types showing different distribution patterns around PD1^high^ and PD1^low^ T cells (mDCs, M1-like M, M2-like M, MDSCs, Fibroblasts, vessels, tumor cells) are highlighted by arrowheads. **(E, F)** Quantification of cells in a 5 µm range around of PD1^high/low^ T cells for tumor margin and tumor core, **(E)** represents immune cells and **(F)** stroma/tumor cells. **(G)** Violin plots for expression levels of eight immune-modulating markers (CD112, CD155, CD276, CD39, CD73, IDO, PD-L1, and VISTA) for the most important immune and tumor cells around PD1^high/low^ T cells in the tumor core area. Violin plots for tumor margin are shown in **Supplementary Figure S5E**. Depicted markers and annotated cell types as indicated by the color code. ROI sizes: Tumor margin (ROI15) and tumor core (ROI16): 975 x 769 µm.

To further reveal the potential for spatial ultra-deep functional phenotyping with our established and validated panel, we demonstrate a comprehensive step-by-step analysis of an HCC sample. We chose three different tumor regions of interest (ROIs) from one patient sample and subsequently analyzed and compared the different tumor regions (**Figure 4**), performed ultra-deep phenotyping of the T cell fraction (**Figures 5**, **6**), and elucidated the T cell neighborhood (**Figure 7**). Selected tumor regions included peritumoral, immune-rich stroma (**Figure 4C**), tumor margin (**Figure 4D**), and tumor core (**Figure 4E**). We further introduced subarea classifications within the different ROIs to describe spatial relationships: Intratumoral stroma (ITS), defined as areas without malignant cells, and intratumoral malignant cell clusters (ITM), defined as areas with densely packed malignant cells. Again, tissue integrity of the specimen after 92 MICS cycles was verified by H&E staining (**Figure 4A**). ROIs were selected based on initial DAPI staining (**Figure 4B**). Advanced cell segmentation resulted in a total of 13006 cells for the stroma, 7552 cells for the tumor margin, and 4917 cells for the tumor core ROI. Tumor stroma was composed of numerous blood and lymphatic vessels, a biliary duct (*white arrow*, top right corner), and a high percentage of immune cells, particularly T cells, PCs, mDCs, and MΦ, organized in clusters without distinguishable B and T cell zone as seen in canonical TLSs (**Figure 4C**). Approaching the tumor site, we identified a collagen- and fibronectin-rich stroma layer with a spotted T cell infiltrate (**Figure 4D**). Interestingly, we found a densely packed immune infiltrate of predominantly myeloid cells at the tumor border region (**Figure 4D**). These myeloid cell populations, dominated by mDCs and MΦ, form a barrier-like structure surrounding the malignant cells (**Figure 4D**). We also identified peritumoral T cell clusters further characterized below. mDCs, MΦ, and T cells are clustered not only at the tumor margin but also in the collagen-rich ECM ITS areas within the tumor core (**Figure 4E**). In contrast, most immune cells are strongly excluded from ITM areas, with only a few T cells and MDSCs being able to infiltrate (**Figures 4D, E**). Expression profiles of 36 tumor-specific markers were compared for tumor margin and border. Violin plots as well as staining showed uniform expression of HCC-related markers like AFP or Glypican 3, as well as pro-oncogenic CD155 and CD36, which are associated with disease progression and poor prognosis in HCC (57, 58) (**Supplementary Figure S3B** and **Supplementary Figures S4A, B**). Quantification of the different cell populations within the three ROIs is provided in **Figure 4F**. In sum, this set of data demonstrates spatially restricted intratumoral heterogeneity in cellular and ECM composition. We show compartmentation and spatial exclusion of certain immune cell populations, elucidating their functional properties as shown below.

### Characterization of T cell subtypes at tumor margin and tumor core

To further demonstrate the potential of the 121 antibody immunophenotyping panel, we next analyzed the T cell compartment within the different HCC tumor ROIs. T cells can be roughly categorized into CD4^+^ T_h_ and CD8^+^ T_c_. Among CD4^+^ T_h_, FoxP3^+^/CD25^+^ regulatory T cells (T_reg_) play a crucial role in orchestrating the immune system, mediating immunosuppressive function within the TIME. We first identified CD45^+^/CD3^+^ T cells in two different HCC tumor regions, namely margin and core. Next, cells were categorized as T_c_, T_h_, or T_reg_ based on the expression of CD8, CD4 or CD4, FoxP3, and CD25 (**Figure 5A**). Additionally, T cells can be phenotypically and functionally described based on their differentiation state. Along this line, we identified six T cell differentiation states based on deep sub-phenotyping using the expression levels of CR45RA, CD45RO, CD27, CD95, and CD11a (**Figure 5B**): naïve T_N_ cells, stem cell-like memory T_SCM_ cells, central memory T_CM_ cells, effector memory T_EM_, effector memory cells re-expressing CD45RA T_EMRA_, and terminally differentiated effector T_TE_ cells (see gating strategy in **Supplementary Figures S5A, B**). We saw a shift towards more CD4^+^ T_h_ and less CD8^+^ T_c_ in the tumor core compared to the tumor margin. T_EM_ cells, both T_h_ cells and T_c_ cells, are the dominant T cell differentiation state in the tumor margin and peritumoral stroma. In contrast, T_CM_ cells and particularly CD8^+^ T_CM_ cells are predominantly found in the core (**Figure 5A**). Looking at the spatial distribution, T_C_ cells, mainly T_CM_, were found to be the only T cell subset capable of infiltrating into ITM areas (**Figures 5C, D**, *left*). The overall fewer CD4^+^ T_h_ cells (19,1% CD4^+^, 80,9% CD8^+^ of all CD3^+^ T cells) were generally located more distant to tumor cell clusters compared to the CD8^+^ T_c_ cells. Utilizing MICS data for external bioinformatic analyses, we were able to cross-validate T cell phenotyping based on marker expression in an unsupervised way. To demonstrate coherence between methods, we provide expression profiles for decisive markers on pre-gated T cell subtypes in **Figure 5E**, confirming subset affiliation.

### Defining functional T cell states and their spatial distribution

Beyond a phenotypical description of different T cell differentiation states, functional characterization plays an important role in understanding and predicting responses to therapy. ICB has revolutionized the therapy of certain cancer entities, however reliable biomarkers for response prediction remain sparse (59–61). Particularly the functional state, spatial distribution, and cellular neighborhood of T cells are crucial for understanding mechanisms of ICB and other immune modulatory therapies. Dysfunctional, exhausted T cells are characterized by the expression of inhibitory immune checkpoint receptors, including PD1, LAG3, and TIM3. In contrast, activated T cells can be identified by the expression of CD69, CD25, and HLA-DR. We used the strength of MICS and analyzed 16 markers associated with T cell function in tumor margin and core. We identified different activation states of T cells – early (CD69), late (CD25), and very late (HLA-DR) (**Figure 6A**). The late and very late activated T cells were clustered in regions proximal to the ITM and within ITS areas (**Figure 6A**, *green arrows*), while T cells within the tumor core, particularly in ITM areas, showed mostly no activation. Along with activation states, we saw a gradient in the expression of exhaustion markers. In **Figure 6B**, PD1 expression levels are depicted based on quartiles of normalized PD1 expression (0-25% = low, 25-75% = intermediate, 75-100% = high PD1 expression). Highest PD1 expression levels were present on cells infiltrated into ITM areas and on the previously described ITM-proximal immune cell clusters (**Figure 6B**, *blue arrows*). Using MICS, and compared to other technologies, we were able to stain additional immune checkpoints and deep sub-phenotype fractions of T cells which were i) PD1 single positive ii) double positive for PD1 and LAG3 or TIM3, or iii) triple positive T cells expressing PD1, LAG3, and TIM3 (**Figure 6C**). Highest T cell exhaustion states were found in ITM-proximal immune cell clusters (**Figure 6C**, *pink arrows*), while ITM-infiltrating T cells predominantly expressed only PD1. Combining the T cell subset definition (**Figure 5**) with the T cell activation and exhaustion states, we were able to thoroughly profile a multitude of T cell subsets for their functional marker and potential therapeutic target expression (**Figure 6D**). In summary, not being limited by markers and having validated antibodies for functional immunophenotyping allows for a stringent and precise T cell classification, which is fundamental for patient-specific therapeutical decisions.

### Immunological constituents of the T cell neighborhood

The major advantage of spatial biology applications towards sc/snRNASeq or other technologies is spatial resolution. Physiological or disease-relevant cellular function and interaction are often limited to restricted areas within a tissue. To further highlight the power of MICS in combination with our established and validated panel, we performed neighborhood analysis focusing on PD1^high^ and PD1^low^ T cells, two clinically relevant T cell subsets. In detail, we performed a distance analysis in MACS iQ View of our anchor cells, PD1^high^ and PD1^low^ T cells, and characterized proximal cell populations. To investigate the relevance of distance to anchor cells in the context of cell-cell interactions, we applied different radii for our analysis. We found that in a 5 µm range a very distinct neighborhood can be described, whereas at broader ranges the informative value faded (**Supplementary Figure S5C**). We therefore focused our downstream analyses on the 5 µm neighborhood (**Figures 7A–C**). Intriguingly, the immediate cellular neighbors around PD1^high^ T cells in ITS areas were predominantly myeloid cells and among them primarily mDCs, M2-like MΦ, and MDSCs (**Figure 7E**). We saw a tendency towards a higher percentage of mDCs and M1-like MΦ in the margin and MDSCs in the core area (**Figure 7E**). As described above, neighborhoods consisting of PD1^high^ T cells and myeloid cells were sited in spatially restricted clusters in close proximity, but strictly excluded from ITM areas (**Figures 7A–C**). In contrast, PD1^high^ T cells infiltrated into ITM areas were almost exclusively surrounded by malignant or other T cells (**Figures 7A–C**). Looking at PD1^low^ T cells, we did not only find a different spatial distribution within the ROIs but also different cellular neighborhoods (**Figure 7F**). PD1^low^ T cells are more often located in perivasculature and fibroblast-rich niches. Furthermore, we found a spatial association of PD1^low^ T cells with plasma cells (**Figure 7E**). To further understand the functional interaction of neighboring cell populations and to cross-validate findings, we analyzed cell populations in the proximity of PD1^high^ and PD1^low^ T cells for the expression of 12 immune checkpoint or immunomodulatory molecules (**Figure 7G** and **Supplementary Figures S5D, E**). Providing intrinsic validation of data, we found relatively higher expression of PD-L1 on cells in PD1^high^ vs. PD1^low^ T cells neighborhoods, particularly expressed by M2-like MΦ, pDCs, plasma cells, endothelial cells, lymphatics vessels, and tumor cells. A similar tendency was found for CD276 on M2-like MΦ, fibroblasts, endothelial, and malignant cells, CD73 on fibroblasts and endothelial cells, CD155 on M1-like and M2-like MΦ, fibroblasts, endothelial and tumor cells, IDO on M2-like MΦ, pDCs, plasma cells, endothelial cells, and lymphatics as well as VISTA on plasma and endothelial cells (**Figure 7G**). Particularly on vasculature, we found a clear spatial correlation between PD1^high^ T cells and protein expression of IFN-γ regulated genes like PD-L1, CD155, CD276, or IDO, as described individually before (62–65). In contrast, there is a tendency towards higher expression of CD112 on M1-like MΦ and CD155 on pDCs in PD1^low^ T cell neighborhoods (**Figure 7G**). Cells in the neighborhood of PD1^low^ and PD1^high^ cells in the tumor margin (**Figures 7A, B**) showed comparable patterns except for higher expression levels of PD-L1 of pDCs, M1-like MΦ, MDSCs, fibroblasts, and tumor cells in PD1^low^ neighborhoods (**Supplementary Figure S5E**). Together, this set of data demonstrates the capacity of MICS-based spatial biology to functionally dissect tumor tissues at single-cell spatial distribution. We identified multilayered, spatially restricted, and functionally distinct cellular neighborhoods. Comparable to T cells within ITM areas, we found less-activated and less-exhausted cells T cells in perivascular and stromal niches facing inhibitory signals via CD39, CD112, and IDO. We identified areas of dense immune infiltration proximal but strictly excluded from ITM areas. These “battle-grounds” show the highest T cell activation but also exhaustion and are dominated by myeloid cells with a tendency towards, but not restricted to suppressive phenotypes, expressing PD-L1, CD39, IDO, and VISTA. Further, we found a limited number of CD8^+^, mainly PD1^high^ T cells, infiltrating into ITM areas where they are surrounded by PD-L1, CD73, CD155, and CD276 expressing tumor cells. These findings clearly demonstrate the spatially restricted functional heterogeneity of distinct cell populations within the very same tumor sample and highlight the eminent need for comprehensive spatially resolved context analyses to derive clinically relevant conclusions.

## Discussion

Multiplexed tissue imaging is an emerging technology providing new and previously unprecedented insights into cellular architecture, function, and orchestration (21, 22, 24). Analyzing the tumor and its TIME with multiplexed tissue imaging techniques allows unprecedented analysis of tumor composition, intra- and intertumoral heterogeneity, immune cell contributions, and the patient-specific therapeutic landscape (20, 25, 27). Here, we present an immunophenotyping panel for ultra-deep spatial profiling of cancerous tissues and the associated TIME. Moreover, we provide an end-to-end workflow for MICS, covering antibody cross-validation, advanced segmentation, marker-based cell annotation, and cellular neighborhood analysis. Analyses for other multiplexed imaging technologies are often based on complex bioinformatic pipelines (66, 67). Our analysis pipeline combines the benefits of the accompanying software for MICS, MACS iQ View, with user-friendly, yet meaningful bioinformatic evaluation in R. We demonstrate and provide templates for advanced cell segmentation and cell type annotation as well as for expression profiling and neighborhood analyses conducted in MACS iQ View without the need of bioinformatic knowledge, which will be useful for many users. We describe MICS as a novel technology to overcome panel-specific limitations of other multiplexed approaches like 1) panel size, 2) complexity of panel design, or 3) antibody cross-reactivity. The established MICS panel was developed based on the following criteria: First, we chose more than one marker for cell type identification and annotation. Since we were interested in identifying the entirety of cells in our samples in contrast to cellular subsets (68, 69), the panel size amounted to more than 120 markers. A major benefit of having such an encompassing panel for multiplexed imaging is the possibility of cross-validating staining and, thereby, increasing the accuracy and confidence of imaging data and cell type annotation. Second, after identification of a cell type, our goal was to comprehensively characterize the functional cellular states in a holistic manner, as compared to other studies which, e.g., only focused on immunoregulatory proteins (25). Therefore, we included not only cell cycle, activation, or differentiation but also cellular stress, exhaustion, and immune-modulating markers, to be able to spatially resolve the complex state of cell types and not only to describe their presence. Third, panel design is much easier for MICS compared to other techniques and no complicated or costly conjugation steps or antibody preparation steps are needed (12, 24, 47, 70, 71). Despite the flexibility and compatibility of antibodies for MICS, we identified antibodies that in our hands, did not fulfill the validation criteria for MICS and summarized them as a resource for other users. To mitigate the issue of false-positive staining due to spectral overlap of FITC and PE, panel and cycle design should ensure that FITC- and PE-labeled antibodies targeting the same cell type/state are not utilized within the same cycle. Moreover, we recommend refraining from integrating FITC-conjugated antibodies targeting strongly expressed antigens in the same cycle as PE-labeled antibodies targeting weakly expressed antigens. Integrating validated APC-conjugated antibodies provided in **Table 1** improves and facilitates individualized panel design. When selecting fluorophores, researchers must consider their varying photostabilities. As MICS relies on photobleaching to allow for the iterative application of the same fluorophores, photo-instable fluorophores have to be used. This can result in acquisition bleaching artifacts, especially for less photostable fluorochromes like FITC and in overlapping regions used for FoV stitching based on DAPI signal (compare **Supplementary Figure S1** and **Supplementary Figure S3C**). Even if these artifacts are present in the individual marker staining images, correct cell type identification was not influenced by these artifacts due to strategic panel design, marker multiplexing for gating, and usage of the visual control in MACS iQ View.

As a proof of concept, we describe the protein expression landscape of the tonsil in an unprecedented way, giving an extensive overview of immune cell subsets and their location. Other studies used tonsils as reference tissues before (26, 72), but with smaller panels or at lower spatial resolution (53). We were able to detect and annotate not only common cell types by using the power of having many lineage-specific markers but also functional subpopulations like CD21^+^ germinal center B cells or CD11b^+^ B cells. Next, we used our panel plus the established analytic workflow for the comprehensive immunological characterization of distinct cancer tissues. Tumors can be categorized in distinct “immunotypes”: 1^st^ immune inflamed, 2^nd^ immune excluded, and 3^rd^ immune desert. These immunotypes have, respectively, been defined as tumors highly infiltrated with immune cells, tumors where T cell infiltrate is limited to tumor stroma and excluded from tumor parenchyma, and tumors that do not exhibit any immune infiltrate (73). Following this framework, we analyzed different primary human cancer samples. We could demonstrate the different tumor architectures with multiplexed imaging concordant with literature for CRC and PCa (9, 66) and show for the first time, to the best of our knowledge, for CCC. We were able to identify and spatially map major immune cell populations. While absolute proportions of immune cells were similar, we found dramatic differences in spatial distribution, underscoring the vast differences in the TIME architecture across different tumor types and different areas within the same tumor samples. We found significant T cell content in all three samples. In the PCa sample, T cells are scattered with direct colocalization to malignant cells, representing an “inflamed-like” immunotype. In contrast, T cells in the CCC sample are spatially segregated from malignant cells by collagen-rich stroma, representing an “excluded-like” immunotype. Both inflamed and excluded areas were found in the CRC sample, suggesting an oversimplification of the classical immunotype model and strengthening the argument that intratumoral heterogeneity can also be described spatially for the immune infiltrate, not only for tumors themselves (74). Analyzing the spatial occurrence of immune cells, we detected and phenotyped TLS in the CRC and CCC samples. The presence of TLS has been put into context with positive immunoreactivity and favorable clinical outcomes (75–77). Since TLS are spatially highly organized structures (78), they can only be identified and described in a spatial context, highlighting the superiority of spatially resolved single-cell technologies.

To further push the limits of ultra-deep spatial immune phenotyping and showcase the power of 120+ marker panels, we next focused on comprehensive functional T cell characterization. HCC, a well-studied cancer type (79–81), served as a reference tissue for our end-to-end workflow testing. We characterized and annotated cell types consistent with literature (81) (**Figure 4**), categorized T cell differentiation, activation, and exhaustion states in ITS and ITM areas (**Figures 5**, **6**), and spatially dissected the cellular neighborhood (**Figure 7**). A comprehensive understanding of T cell function and dysfunction within the TIME is crucial to predict responses to immunotherapies, particularly ICB, stratify patients, and identify novel target structures (73). To the best of our knowledge, we describe for the first time the *in situ* characterization of more than 20 differentiation and functional T cell phenotypes (82, 83), including markers for T cell activation (CD69, CD25, HLA-DR) (73, 84, 85) and exhaustion (PD1, LAG3, TIM3, CTLA-4) (86–89). Although this study was not designed to draw general conclusions for a specific tumor entity, our comprehensive data set allowed extensive observations suitable to be transferred to bigger tumor cohorts. The analyzed HCC sample was classified as an “excluded” immunotype. In line with previous studies, we describe a dense deposition of collagen-rich ECM at the tumor margin and ITS area (90). Within these areas, predominantly CD8^+^ T_CM_ cells, associated with beneficial outcomes in HCC (83), are colocalized with myeloid cells, particularly mDCs and M2-like MΦ. Applying neighborhood analysis, we describe multiple distinct immune ecosystems within the same tumor sample. These organized cellular neighborhoods, composed of various T cell subsets, myeloid, and stromal cells, have been proposed to regulate anti-tumor T cell responses and, consequently, response to therapy and patient survival (73, 91–93). Evidently, a comprehensive understanding of the spatial neighborhoods and cellular crosstalk is indispensable to identify novel biomarkers and therapy targets or combinations thereof. While previous reports focused on specific aspects, either specific cell populations, spatial relations thereof, or distinct immunomodulatory molecules of the HCC TIME (79, 80, 94–96), our 120+ marker panel provides a holistic view. As demonstrated for PD1^high^ T cells, ultra-deep phenotyping identifies not only spatially co-localized cell populations but rather allows for the functional description of immune ecosystems. We show multiple distinct PD1^high^ T cell associated hubs consisting of different PD-L1 positive neighboring cell populations and provide co-expression profiles of a plethora of immune regulatory proteins influencing T cells (79, 81, 96, 97). Together this exemplary analysis, focused on T cell phenotyping and neighborhood analysis, clearly demonstrates the potential of our 120+ marker MICS panel and analytic workflow and could be easily expanded to other relevant cell types and functional subpopulations of the TIME.

With this study, we intend to provide a blueprint for next-generation ultra-deep spatial tissue profiling using MICS technology. MICS allows unlimited flexibility in panel design, both in terms of antibody availability and panel size. To support researchers, we provide lists of validated antibodies applicable for MICS. In our hands, MICS is a user-friendly and reliable technology facilitating easy access to spatial biology applications even for inexperienced users. Further, we describe a validated analytic workflow based on the provided software MACS iQ View and R. While this feasibility study was not powered for clinically relevant discoveries, the presented panel and analytic workflow indeed allow for massive parallel monitoring of highly complex tumor tissues, thereby covering malignant cells, ECM composition, stroma cells, vasculature, and immune cells. Furthermore, cellular states can be defined (e.g., proliferation, activation, stress, or exhaustion), immune targets and immune modulatory molecules can be monitored as well as distinct immune ecosystems. The unprecedented spatial analysis depth using multiplexed tissue imaging can be applied to numerous immuno-oncological research questions, pathing the way to a holistic understanding of the TIME in solid tumors and promoting precision immunotherapy.

## Data availability statement

The original contributions presented in the study are included in the article/**Supplementary Materials**. Data and code used for analysis in this study are deposited at: Zenodo: 10.5281/zenodo.10057717. Further inquiries can be directed to the corresponding author/s.

## Ethics statement

The studies involving humans were approved by Ethics Committee of the University Hospital Tübingen (ethics approval No. 508-2016BO1). The studies were conducted in accordance with the local legislation and institutional requirements. The participants provided their written informed consent for biobanking and the use of biomaterials and clinical data for scientific assessment.

## Author contributions

SS: Conceptualization, Formal analysis, Investigation, Methodology, Supervision, Visualization, Writing – original draft, Writing – review & editing. BK: Formal analysis, Visualization, Writing – review & editing. FE: Investigation, Visualization, Writing – review & editing. AN: Investigation, Visualization, Writing – review & editing. DS: Formal analysis, Writing – review & editing. MK: Investigation, Writing – review & editing. TA: Resources, Writing – review & editing, Validation. WS: Resources, Writing – review & editing, Validation. CS: Conceptualization, Funding acquisition, Supervision, Writing – original draft, Writing – review & editing.
