# Supplementary material for: Unveiling spatial complexity in solid tumor immune microenvironments through multiplexed imaging
Source: Front Immunol. 2024 Mar 19;15:1383932. doi: 10.3389/fimmu.2024.1383932 (PMC10985204; doi:10.3389/fimmu.2024.1383932)
Supplement: Supplementary file 1 [file Image_1.pdf]

Figure S1

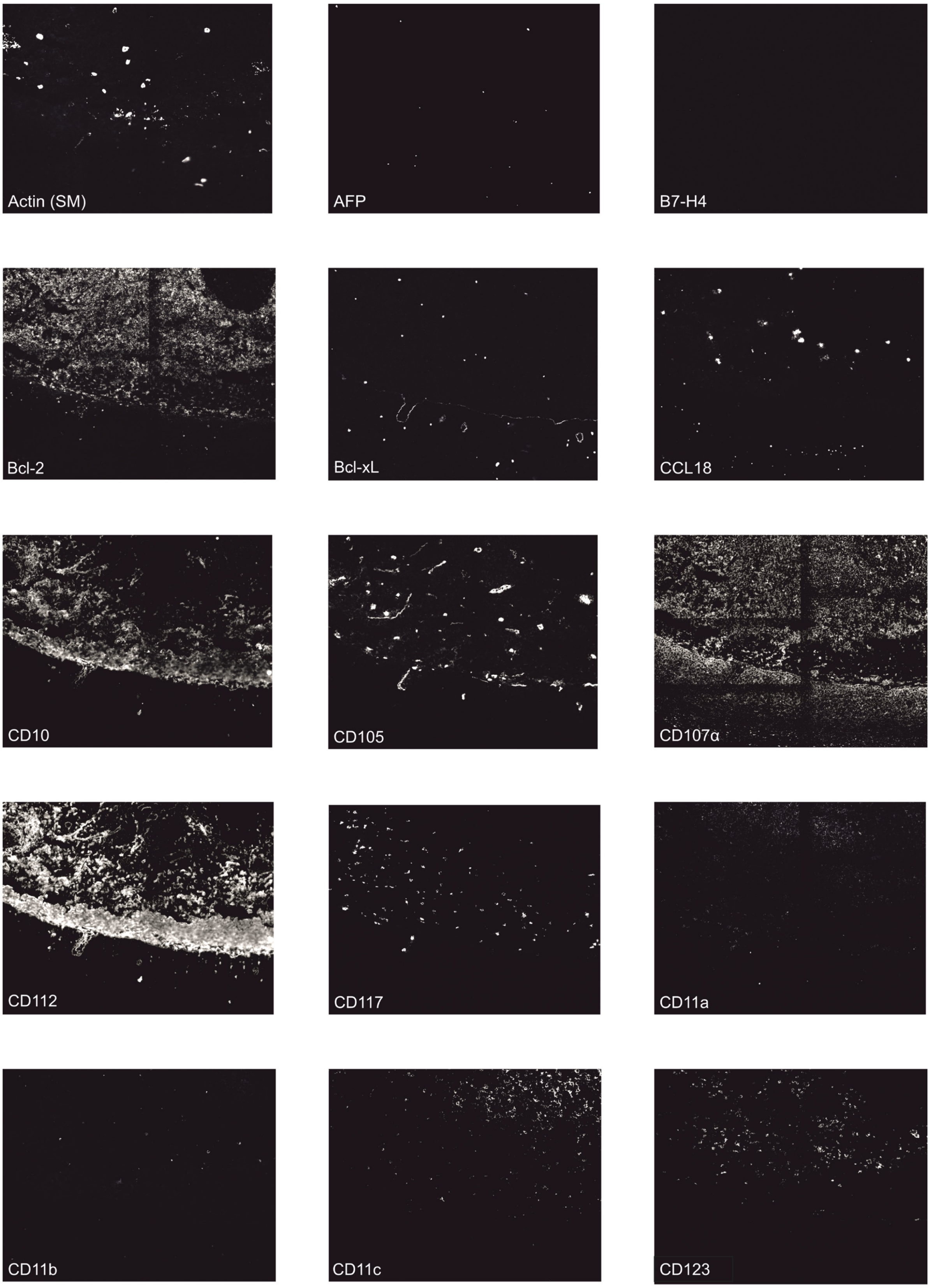
Actin (SM)
AFP
B7-H4
Bcl-2
Bcl-xL
CCL18
CD10
CD105
CD107α
CD112
CD117
CD11a
CD11b
CD11c
CD123

Figure S1

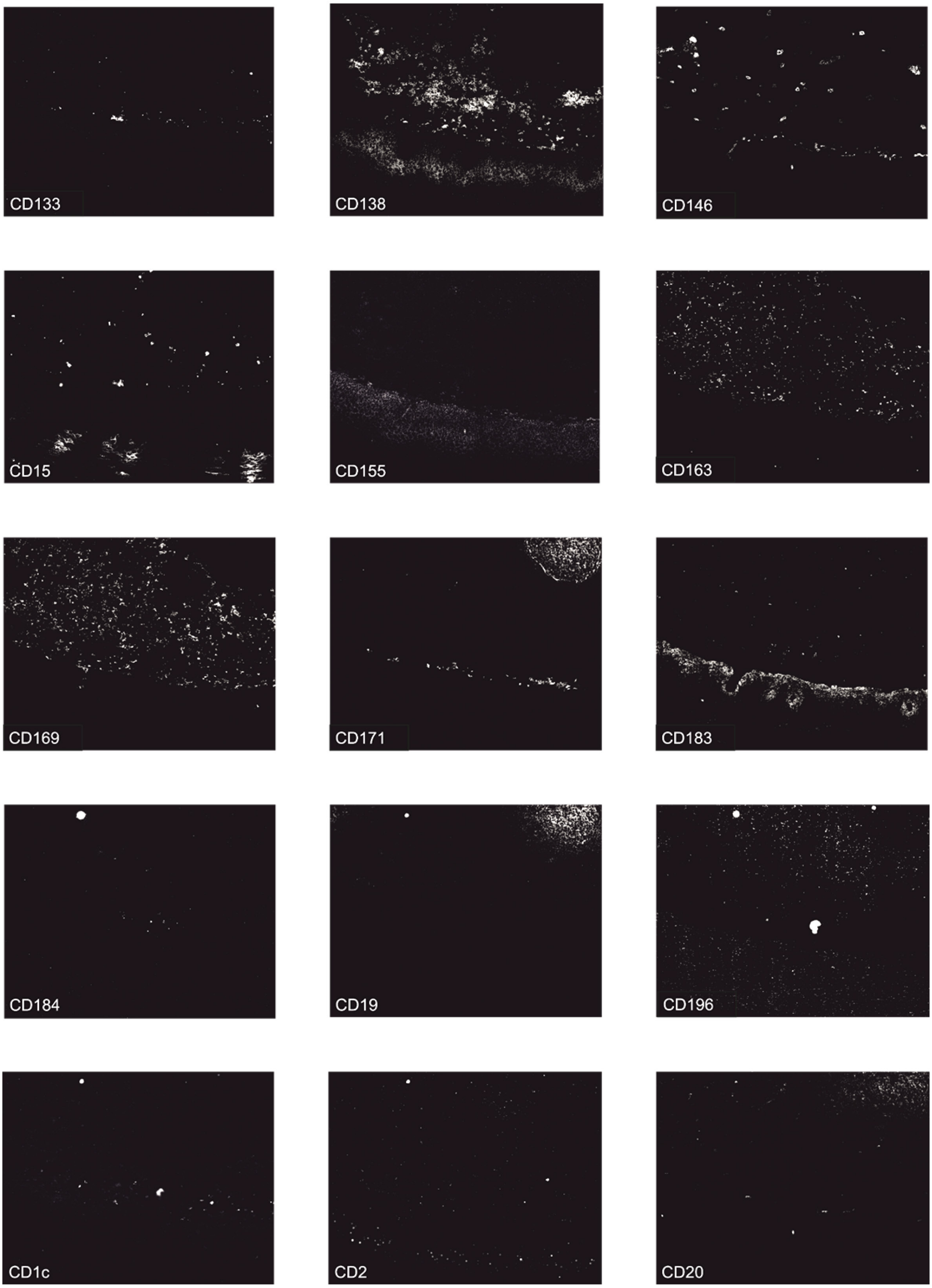
CD133
CD138
CD146
CD15
CD155
CD163
CD169
CD171
CD183
CD184
CD19
CD196
CD1c
CD2
CD20

Figure S1

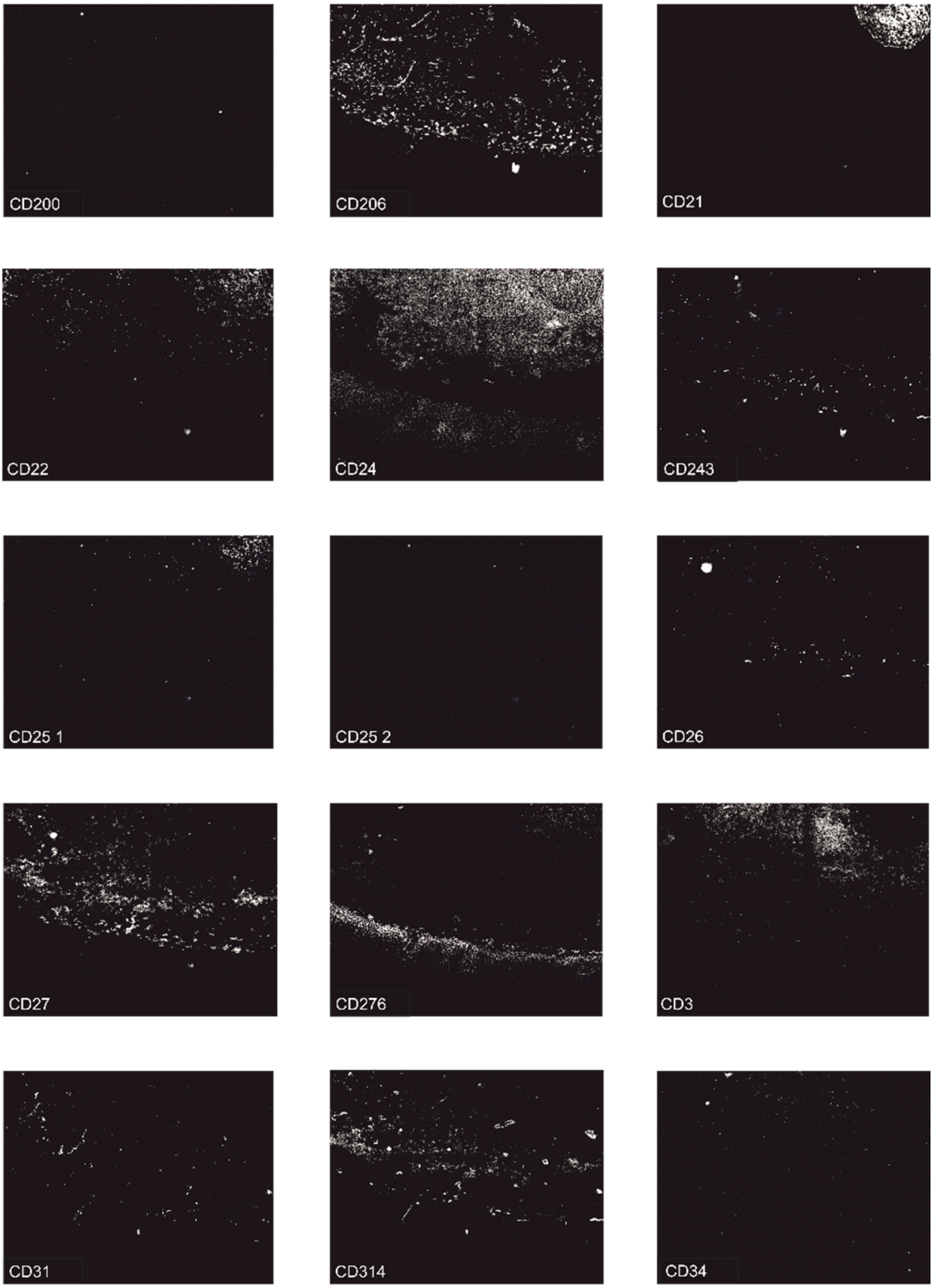
CD200
CD206
CD21
CD22
CD24
CD243
CD25 1
CD25 2
CD26
CD27
CD276
CD3
CD31
CD314
CD34

Figure S1

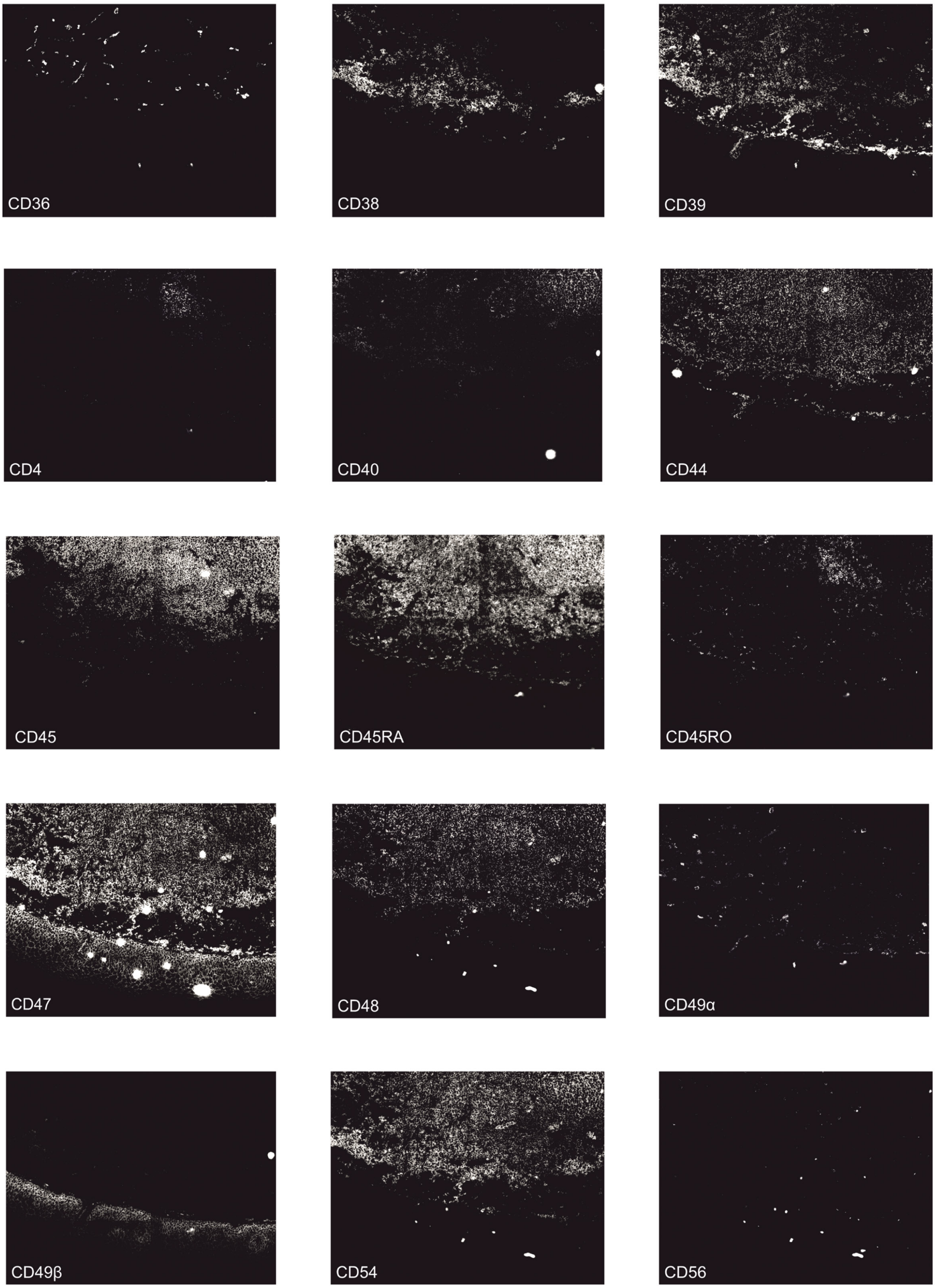
CD36
CD38
CD39
CD4
CD40
CD44
CD45
CD45RA
CD45RO
CD47
CD48
CD49α
CD49β
CD54
CD56

Figure S1

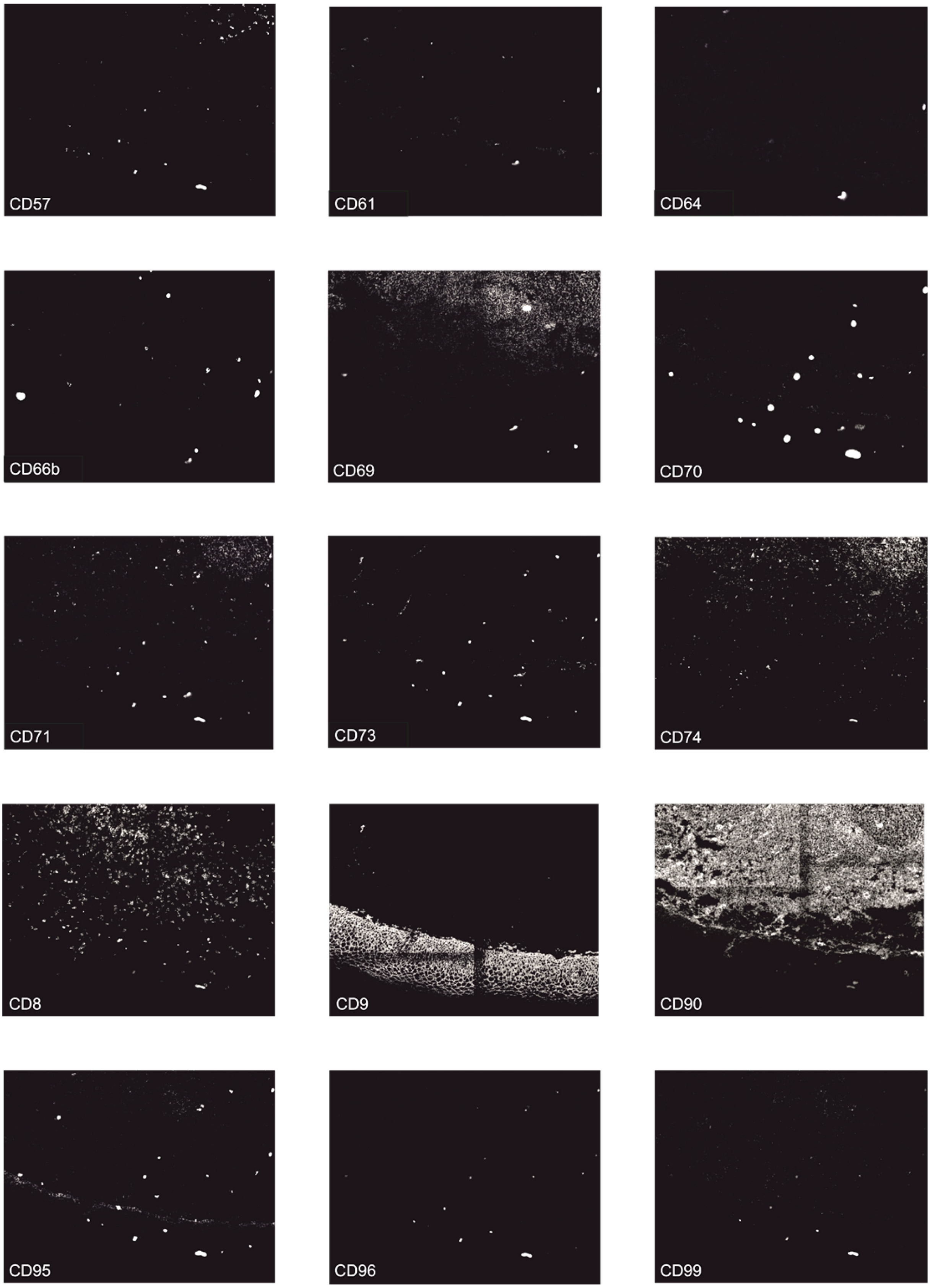
CD57
CD61
CD64
CD66b
CD69
CD70
CD71
CD73
CD74
CD8
CD9
CD90
CD95
CD96
CD99

Figure S1

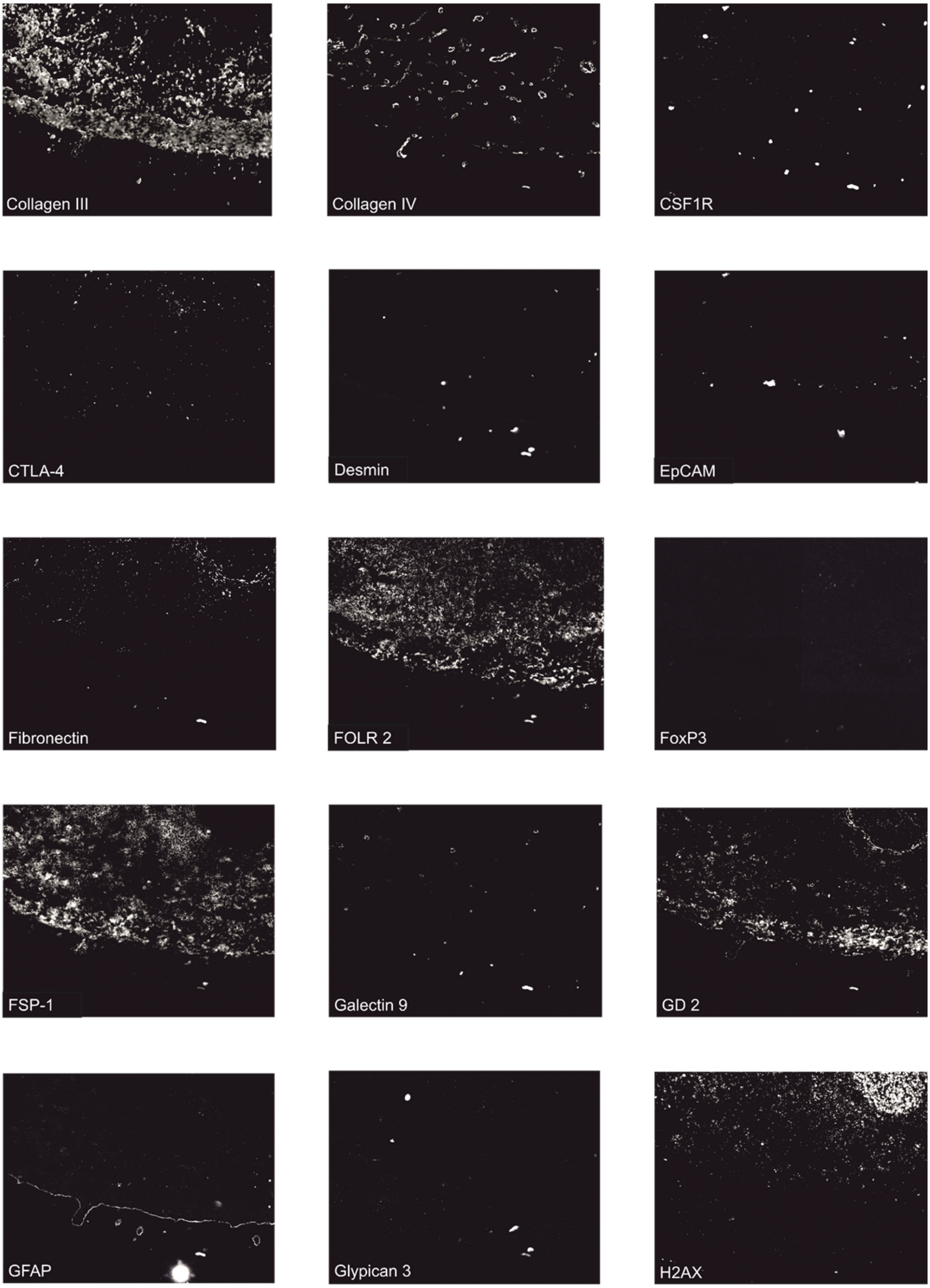
Collagen III
Collagen IV
CSF1R
CTLA-4
Desmin
EpCAM
Fibronectin
FOLR 2
FoxP3
FSP-1
Galectin 9
GD 2
GFAP
Glypican 3
H2AX

Figure S1

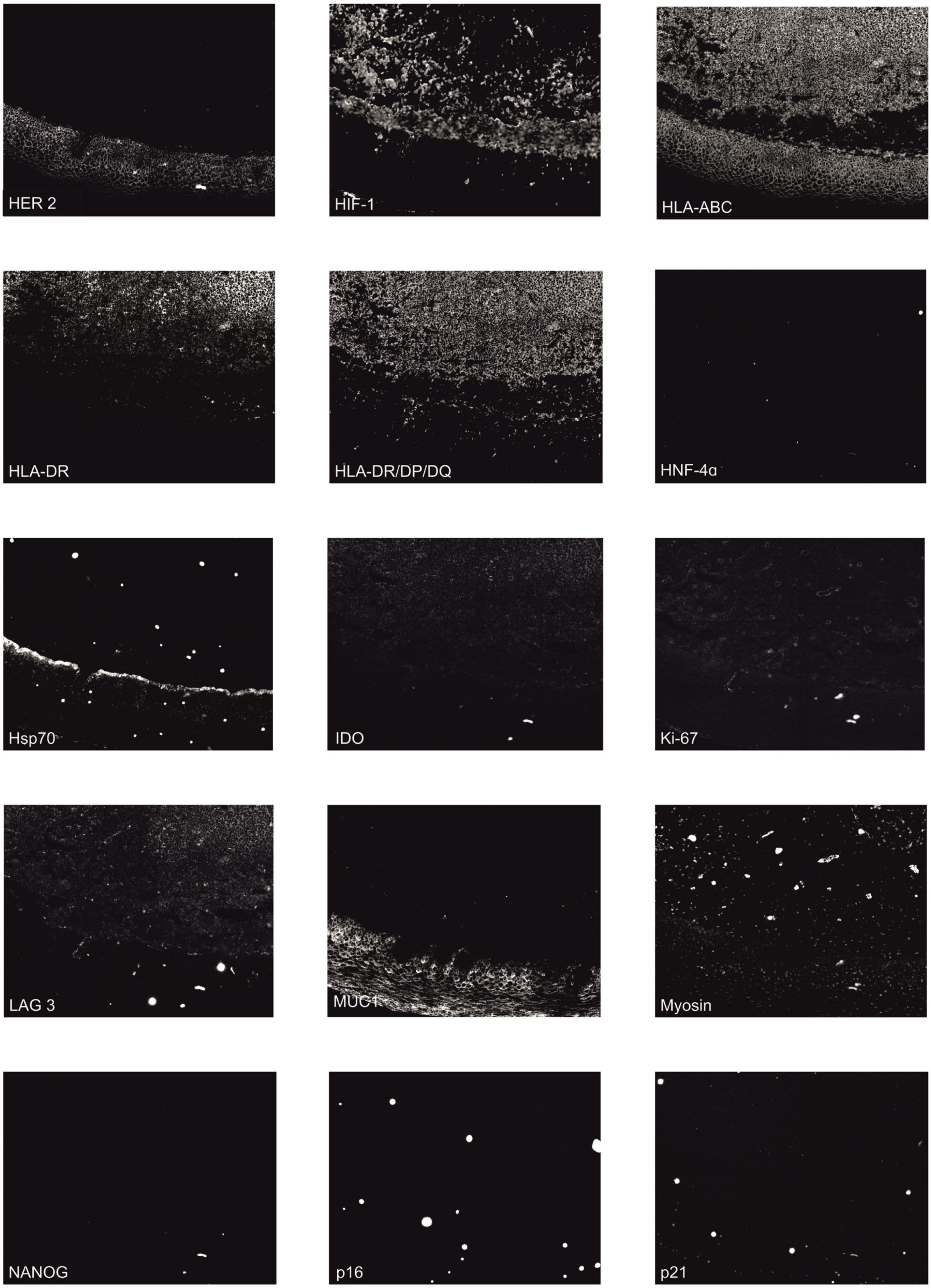
HER 2
HIF-1
HLA-ABC
HLA-DR
HLA-DR/DP/DQ
HNF-4α
Hsp70
IDO
Ki-67
LAG 3
MUC1
Myosin
NANOG
p16
p21

Figure S1

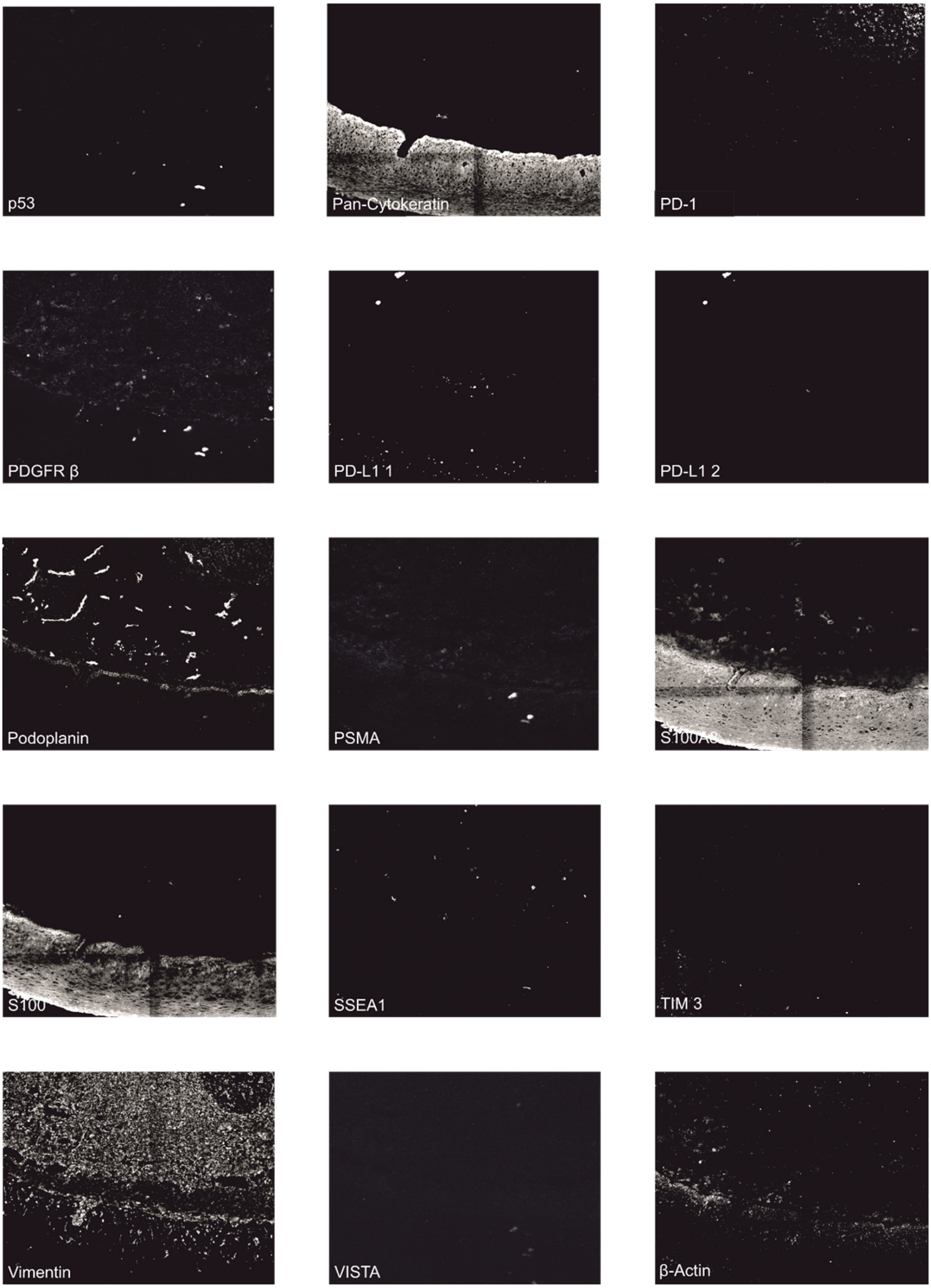
p53
Pan-Cytokeratin
PD-1
PDGFR β
PD-L1 1
PD-L1 2
Podoplanin
PSMA
S100A
S100
SSEA1
TIM 3
Vimentin
VISTA
β-Actin

Figure S1

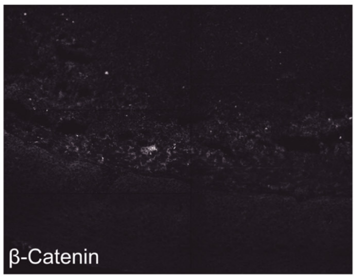

Supplementary Figure 1: Single staining of immunophenotyping panel on human tonsil tissue. 121 single staining images of the immunophenotyping panel are depicted.

ROI sizes: 976 x 769 μm
