# Supplementary material for: Unveiling spatial complexity in solid tumor immune microenvironments through multiplexed imaging
Source: Front Immunol. 2024 Mar 19;15:1383932. doi: 10.3389/fimmu.2024.1383932 (PMC10985204; doi:10.3389/fimmu.2024.1383932)
Supplement: Supplementary file 2 [file Image_2.pdf]

Figure S2

A

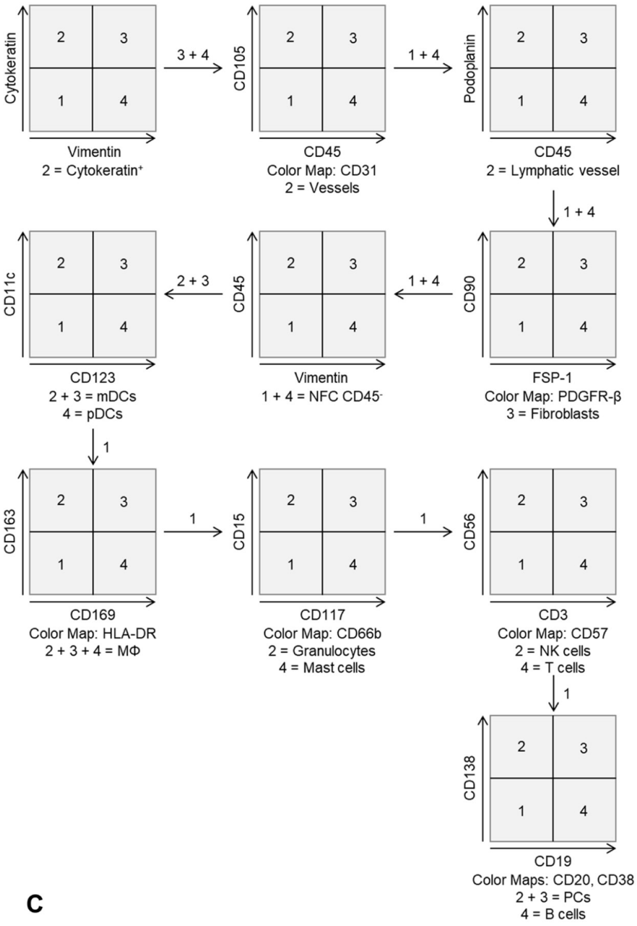

B

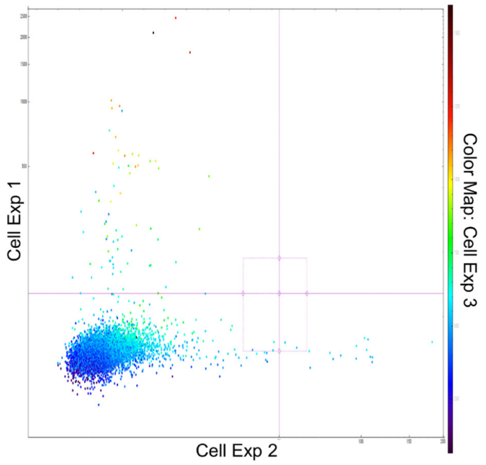

C

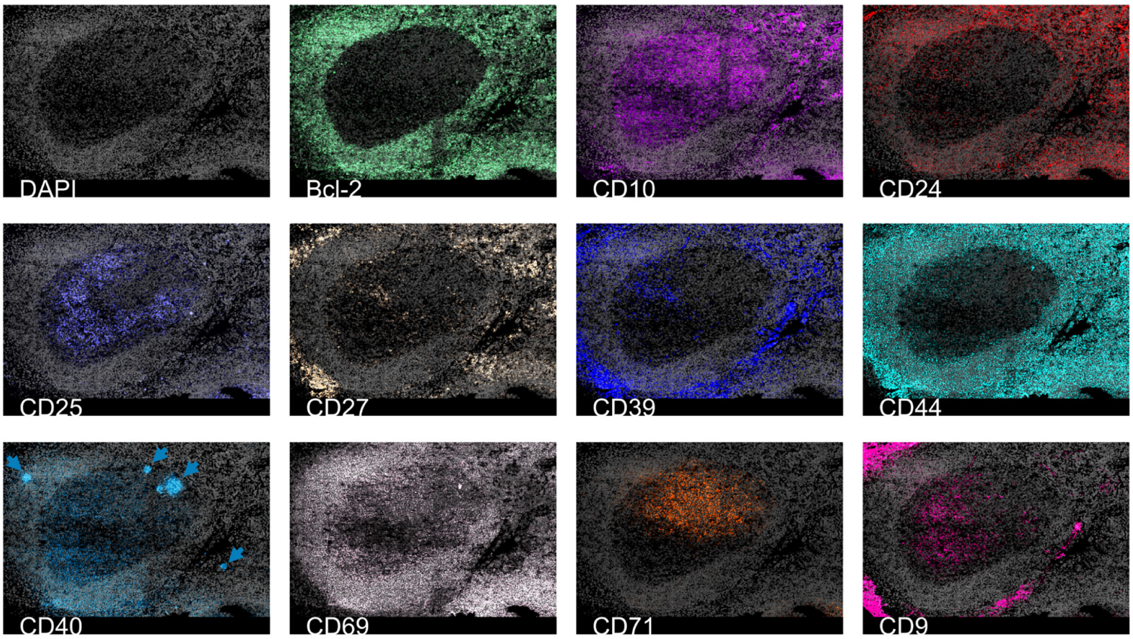

Supplementary Figure 2:

Cell type annotation and sub-phenotyping of human tonsil tissue. A Expert-based gating analysis for immune cells identification which can be applied to segmented cells within MACSiQ View or externally (with exported FCS files). **B** Showing the Color Map function in MACSiQ View which allows for multiparametric gating with more than two markers. **C** Marker expressions used for tonsil sub-phenotyping: mantle zone: $CD27^+$, $CD39^+$; GC: $CD9^+$, $CD10^+$, CD171+; non-GC: Bcl-2^+, $CD44^+$, B cell activation states ($CD25^+$, $CD44^+$, $CD69^+$) and B cell memory phenotype ($CD40^+$).

ROI sizes: 976 x 640 µm
