# Supplementary material for: Unveiling spatial complexity in solid tumor immune microenvironments through multiplexed imaging
Source: Front Immunol. 2024 Mar 19;15:1383932. doi: 10.3389/fimmu.2024.1383932 (PMC10985204; doi:10.3389/fimmu.2024.1383932)
Supplement: Supplementary file 3 [file Image_3.pdf]

Figure S3

A

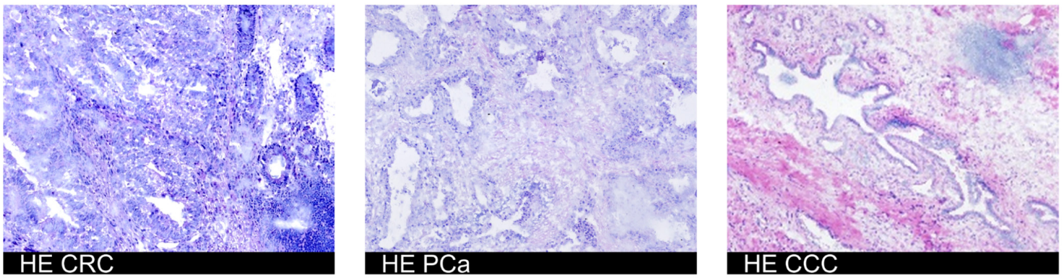

HE CRC
HE PCa
HE CCC

B

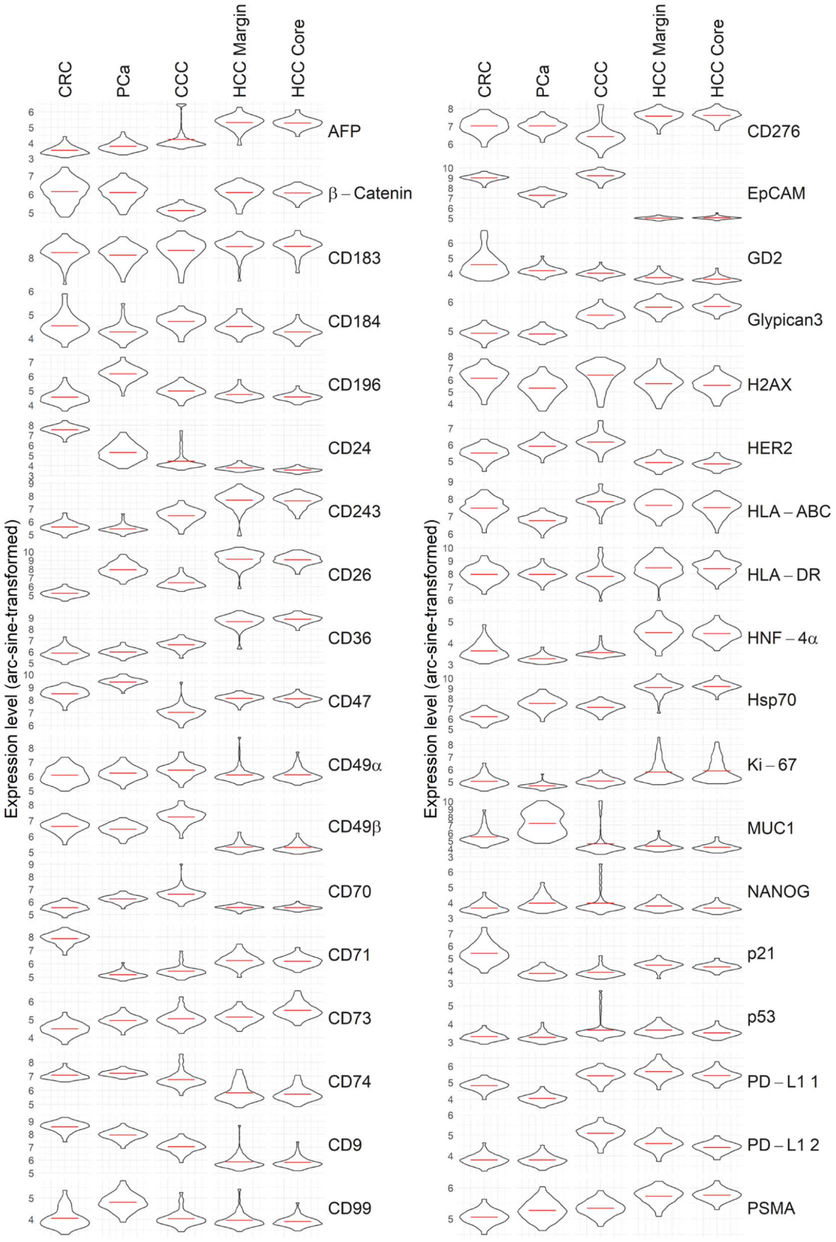

CRC
PCa
CCC
HCC Margin
HCC Core
Expression level (arc-sine-transformed)
AFP
β – Catenin
CD183
CD184
CD196
CD24
CD243
CD26
CD36
CD47
CD49α
CD49β
CD70
CD71
CD73
CD74
CD9
CD99
CD276
EpCAM
GD2
Glypican3
H2AX
HER2
HLA – ABC
HLA – DR
HNF – 4α
Hsp70
Ki – 67
MUC1
NANOG
p21
p53
PD – L1 1
PD – L1 2
PSMA

Figure S3

C

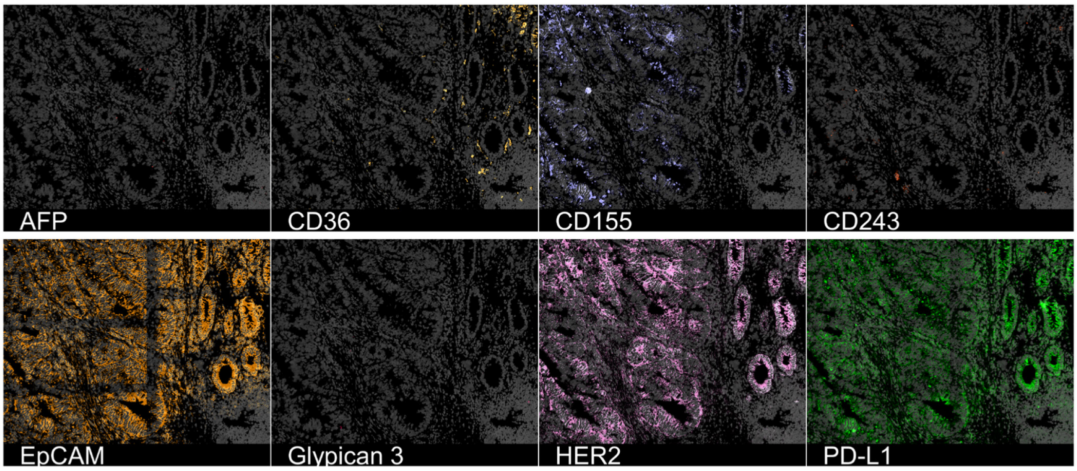

D

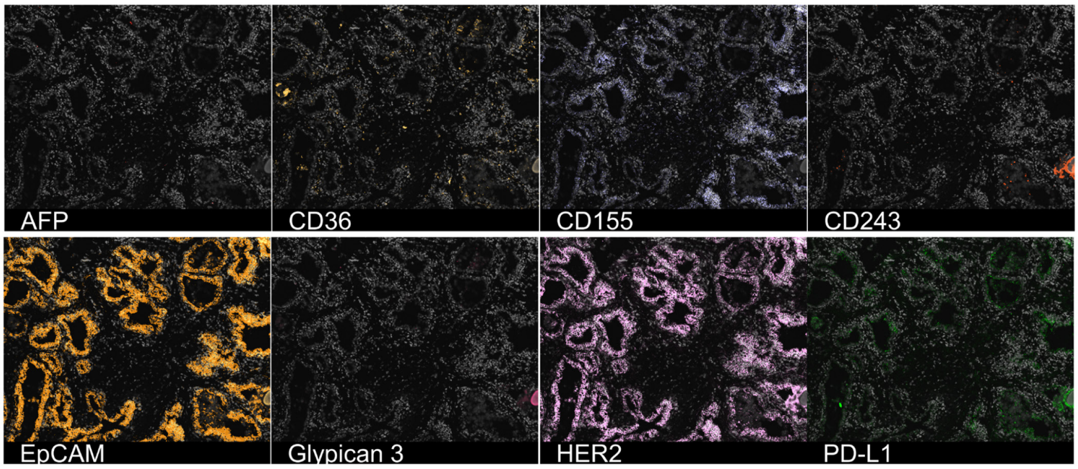

E

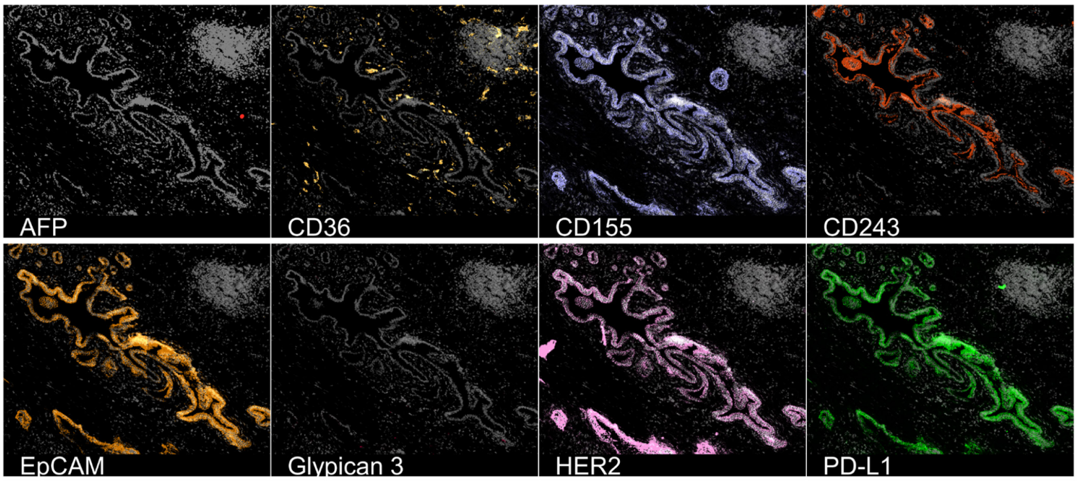

Figure S3

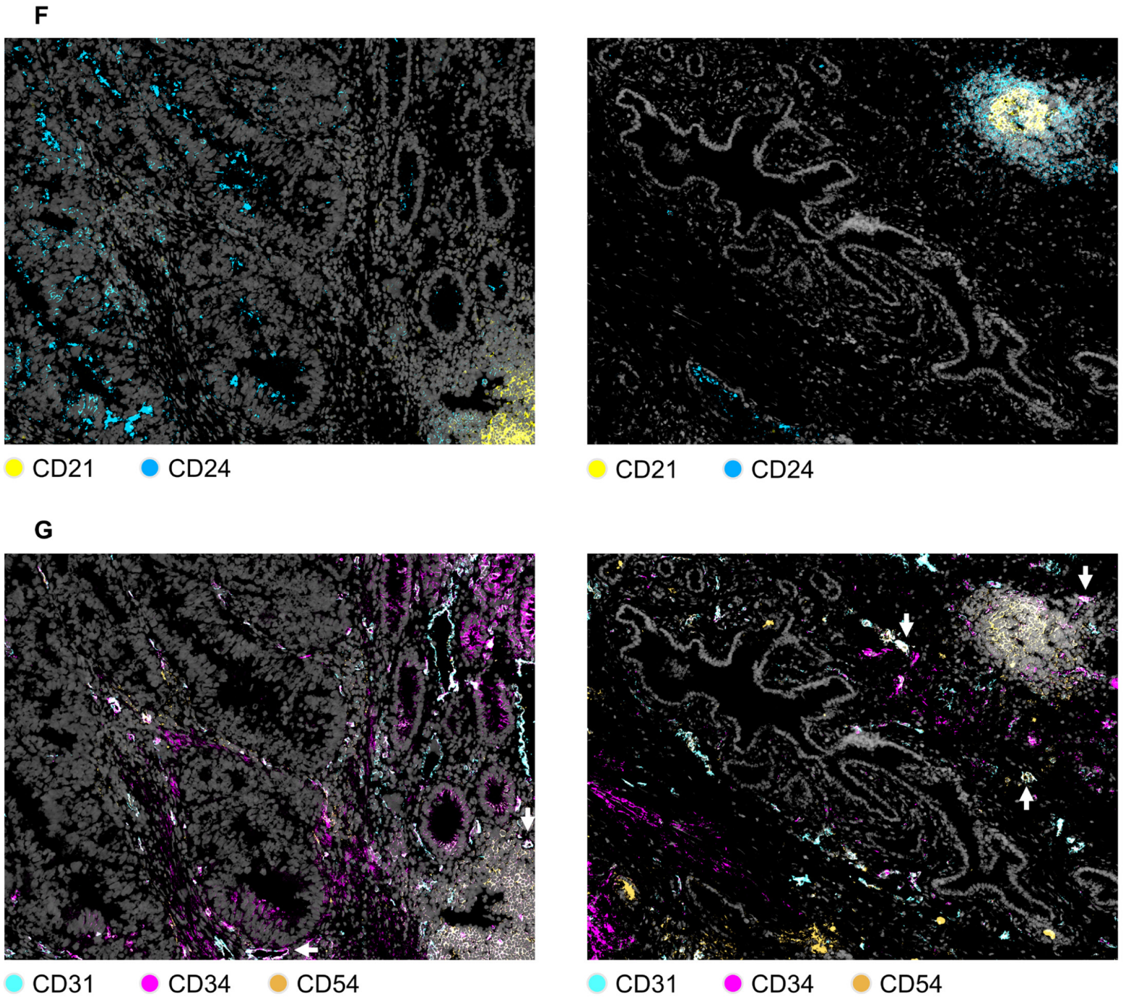

Supplementary Figure 3: Deep spatial phenotyping of CRC, PCa, and CCC malignant, stromal, and immune cells. A H&E staining after 89-92 MICS cycles of CRC, PCa, and CCC. **B** 36 different cancer-associated protein marker expression levels depicted as violin plots, showing the tumor heterogeneity between the analyzed entities. **C-E** Staining of immunotherapeutic targets and tumor markers (AFP, CD36, CD155, CD243, EpCAM, Glypican 3, HER2, and PD-L1) on **C** CRC, **D** PCa, and **E** CCC. **F** TLS with GC reaction, shown by $CD21^{+}$ B cells. **G** HEV formation surrounding the TLS is depicted by $CD34^{+}/CD54^{+}$ endothelial cells and highlighted by white arrowheads.

ROI sizes: CRC: 975 x 747 µm, PCa: 974 x 747 µm, CCC: 974 x 769 µm.
