# Supplementary material for: Unveiling spatial complexity in solid tumor immune microenvironments through multiplexed imaging
Source: Front Immunol. 2024 Mar 19;15:1383932. doi: 10.3389/fimmu.2024.1383932 (PMC10985204; doi:10.3389/fimmu.2024.1383932)
Supplement: Supplementary file 4 [file Image_4.pdf]

Figure S4

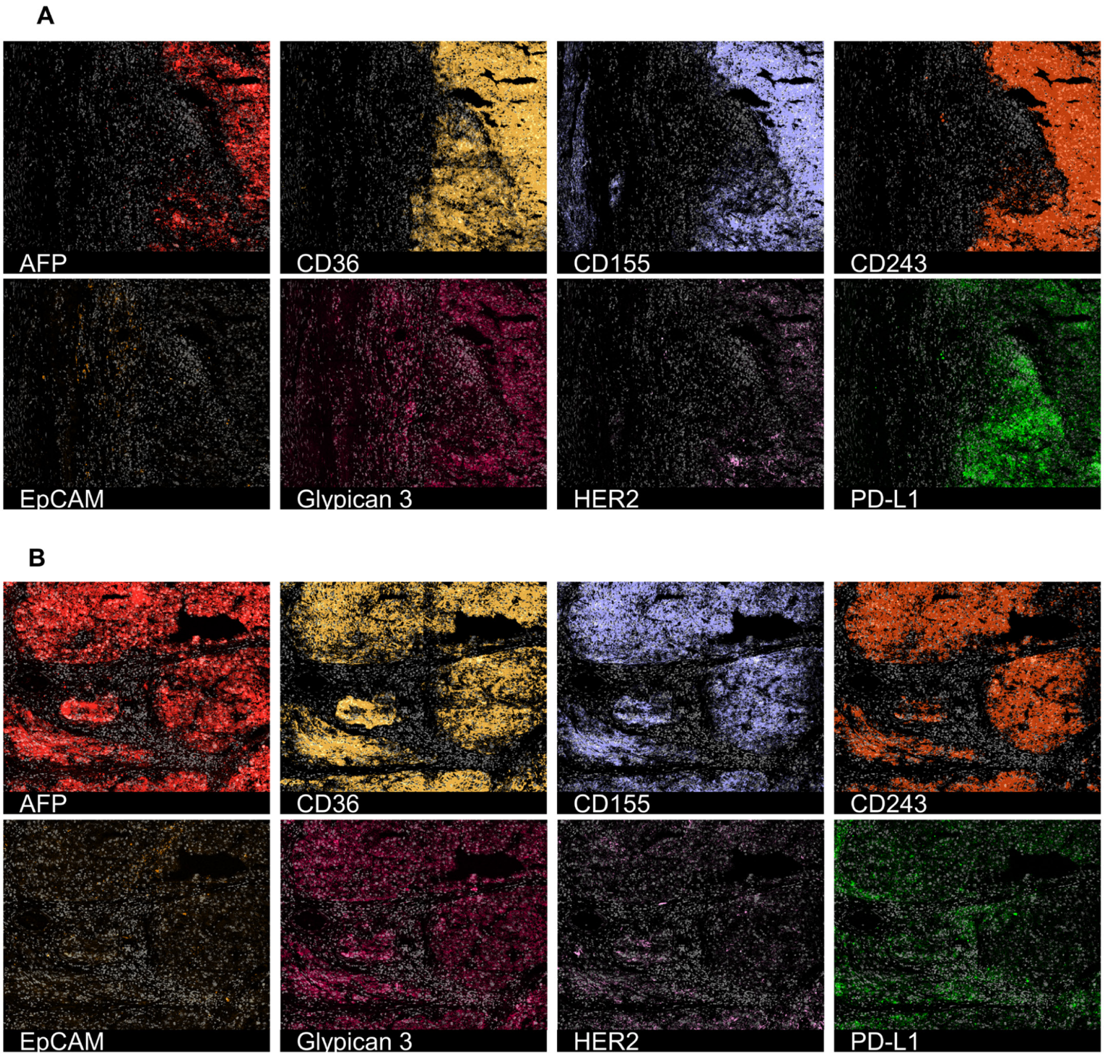

Supplementary Figure 4: Spatial HCC tumor characterization. Staining of immunotherapeutic targets and tumor markers (AFP, CD36, CD155, CD243, EpCAM, Glypican 3, HER2, and PD-L1) on HCC tumor margin (**A**) and tumor core (**B**).

ROI sizes: Tumor margin (ROI15) and tumor core (ROI16): 975 x 769 µm.
