# Supplementary material for: Unveiling spatial complexity in solid tumor immune microenvironments through multiplexed imaging
Source: Front Immunol. 2024 Mar 19;15:1383932. doi: 10.3389/fimmu.2024.1383932 (PMC10985204; doi:10.3389/fimmu.2024.1383932)
Supplement: Supplementary file 5 [file Image_5.pdf]

Figure S5

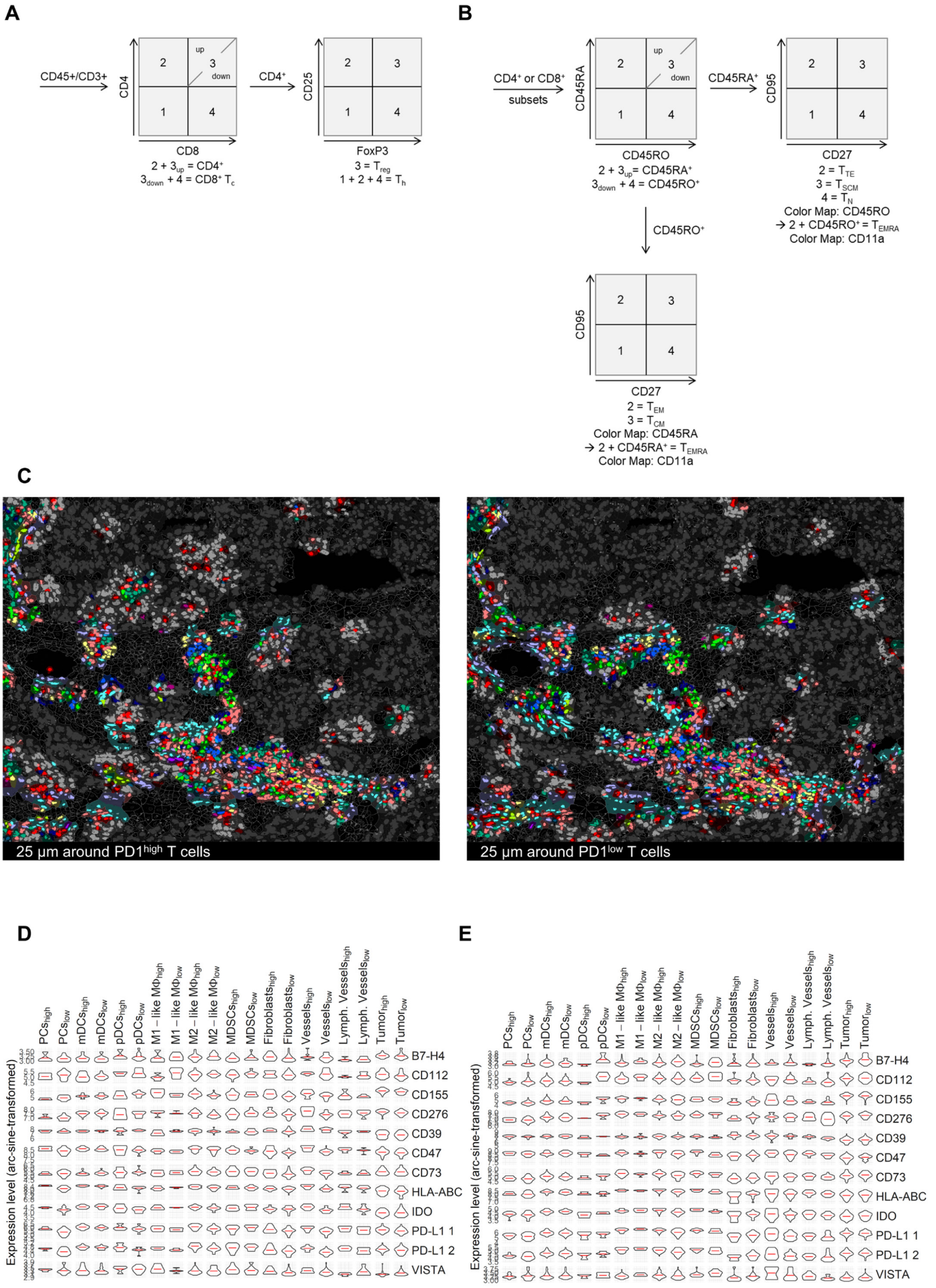
A
CD45+/CD3+
CD4
CD8
up
down
2 + 3up = CD4+
3down + 4 = CD8+ Tc
CD4+
CD25
FoxP3
3 = Treg
1 + 2 + 4 = Th
B
CD4+ or CD8+ subsets
CD45RA
CD45RO
2 + 3up = CD45RA+
3down + 4 = CD45RO+
CD45RA+
CD95
CD27
2 = TTE
3 = TSCM
4 = TN
Color Map: CD45RO
→ 2 + CD45RO+ = TEMRA
Color Map: CD11a
CD45RO+
2 = TEM
3 = TCM
Color Map: CD45RA
→ 2 + CD45RA+ = TEMRA
Color Map: CD11a
C
25 µm around PD1high T cells
25 µm around PD1low T cells
D
E
Expression level (arc-sine-transformed)
PCshigh
PCslow
mDCshigh
mDCslow
pDCshigh
pDCslow
M1 – like MΦhigh
M1 – like MΦlow
M2 – like MΦhigh
M2 – like MΦlow
MDSCshigh
MDSCslow
Fibroblastshigh
Fibroblastslow
Vesselshigh
Vesselslow
Lymph. Vesselshigh
Lymph. Vesselslow
Tumorhigh
Tumorlow
B7-H4
CD112
CD155
CD276
CD39
CD47
CD73
HLA-ABC
IDO
PD-L1 1
PD-L1 2
VISTA

Supplementary Figure 5: T cell differentiation subtype gating scheme and T cell neighborhood analysis in HCC. A Expert-based gating analysis for identification of Th, Tc, and Treg out of a $CD45^{+}/CD3^{+}$ population**. B** Expert-based gating analysis for T cell differentiation subsets using the expression levels of CR45RA, CD45RO, CD27, CD95, and CD11a. Six T cell differentiation subsets were defined: naïve T_{N} cells, stem cell-like memory T_{SCM} cells, central memory T_{CM} cells, effector memory T_{EM}, effector memory cells re-expressing CD45RA T_{EMRA}, and terminally differentiated effector T_{TE} cells. **C** Exemplary neighborhood analysis of $PD1^{high/low}$ T cells in the HCC tumor core by applying a 25 µm range. Due to a bigger range, neighboring cells of both T cells populations overlap and the informative value fades. **D, E** Expression profiles of 12 immune checkpoint or immunomodulatory molecules (B7-H4, CD112, CD155, CD276, CD39, CD47, CD73, HLA-ABC, IDO, PD-L1 1, PD-L1 2, and VISTA) for PCs, mDCs, pDCs, M1-like MΦ, M2-like MΦ, MDSCs, fibroblasts, vessel, lymphatic vessels, and tumor cells in a 5 µm range around $PD1^{high/low}$ T cells in tumor core (**D**) and tumor margin (**E**).

ROI sizes: Tumor margin (ROI15) and tumor core (ROI16): 975 x 769 µm.
