# Supplementary material for: Unveiling spatial complexity in solid tumor immune microenvironments through multiplexed imaging
Source: Front Immunol. 2024 Mar 19;15:1383932. doi: 10.3389/fimmu.2024.1383932 (PMC10985204; doi:10.3389/fimmu.2024.1383932)
Supplement: Supplementary file 6 [file Table_1.docx]

**Table S1: Tested and excluded antibodies.**

| **Antigen** | **Clone** | **Tested**  **Dilutions** | **Order No.** | **Fluorochrome** | **Company** |
| --- | --- | --- | --- | --- | --- |
| A2A-R | 7FG-G5-A2 | 50 | sc-32261FITC | FITC | SantaCruz Biotechnology |
| ALK | F-12 | 50 | sc-398791PE | PE | SantaCruz Biotechnology |
| Arginase-1 | Polyclonal | 100 | NBP1-32731PE | PE | Novus Biologicals |
| CD134 | REA621 | 50 | 130-126-024 | PE | Miltenyi Biotec |
| CD14 | REA599 | 50 | 130-110-518 | FITC | Miltenyi Biotec |
| CD16 | 3G8 | 200, 100, 50 | 302006 | FITC | Biolegend |
| CD181 | REA958 | 50 | 130-115-878 | FITC | Miltenyi Biotec |
| CD185 | REAL284 | 50 | 130-115-976 | FITC | Miltenyi Biotec |
| CD273 | REA985 | 50 | 130-116-563 | PE | Miltenyi Biotec |
| CD274 | 29E.2A3 | 200, 100, 50 | 329706 | PE | BioLegend |
| CD277 | BT3.1 | 50 | 130-117-693 | PE | Miltenyi Biotec |
| CD28 | REAL105 | 50 | 130-112-078 | FITC | Miltenyi Biotec |
| CD33 | 44M12D3 | 200 | NBP2-22377F | FITC | Novus Biologicals |
| CD87 | REA892 | 50 | 130-114-849 | FITC | Miltenyi Biotec |
| CKR4 | G2 | 50 | sc377357PE | PE | SantaCruz Biotechnology |
| CSF1R | 34A4 | 50 | sc02PE | PE | SantaCruz Biotechnology |
| CX3CR1 | REA385 | 50 | 130-122-912 | PE | Miltenyi Biotec |
| CXCR4 | 4G10 | 50 | sc-53534FITC | FITC | SantaCruz Biotechnology |
| EGFR | REA688 | 50 | 130-110-528 | PE | Miltenyi Biotec |
| FGFR4 | REA1243 | 50 | 130-124-911 | PE | Miltenyi Biotec |
| FR | E-11 | 50 | sc-515521PE | PE | SantaCruz Biotechnology |
| Glypican2 | F-5 | 50 | sc-393824PE | PE | SantaCruz Biotechnology |
| IL1RAcP | D-5 | 50 | sc-376872PE | PE | SantaCruz Biotechnology |
| KIR2D | REA1042 | 50 | 130-117-477 | FITC | Miltenyi Biotec |
| SLUG | 666633 | 50 | IC7408P | PE | RnD Systems |
| TGFbRII | REA903 | 50 | 130-115-023 | FITC | Miltenyi Biotec |
| TIGIT | REA1004 | 100 | 130-116-814 | PE | Miltenyi Biotec |
| TrkA | REA430 | 50 | 130-117-705 | PE | Miltenyi Biotec |
| WT1 | REA925 | 50 | 130-115-470 | PE | Miltenyi Biotec |
