# Supplementary material for: Unveiling spatial complexity in solid tumor immune microenvironments through multiplexed imaging
Source: Front Immunol. 2024 Mar 19;15:1383932. doi: 10.3389/fimmu.2024.1383932 (PMC10985204; doi:10.3389/fimmu.2024.1383932)
Supplement: Supplementary file 7 [file Table_2.docx]

| **Table S2: Patient characteristics.** | | |  |  |  |
| --- | --- | --- | --- | --- | --- |
| **Patient ID** | **Age** | **Gender** | **Diagnosis** | **Disease stage** | **Neoadjuvant therapy** |
| 1 | 64 | m | CRC | G1 | - |
| 2 | 46 | w | CCC | G1 | - |
| 3 | 60 | m | PCa | G2b (Gleason 7b) | - |
| 4 | 73 | m | HCC | G3 | TACE |
