# Supplementary material for: Unveiling spatial complexity in solid tumor immune microenvironments through multiplexed imaging
Source: Front Immunol. 2024 Mar 19;15:1383932. doi: 10.3389/fimmu.2024.1383932 (PMC10985204; doi:10.3389/fimmu.2024.1383932)
Supplement: Supplementary file 8 [file Table_3.docx]

| **Nucleus** | | | | | | **Cytoplasm** | | | | **Applied to which tissue:** |
| --- | --- | --- | --- | --- | --- | --- | --- | --- | --- | --- |
| Method | Reference channel | Detection sensitivity [%] | Min/max Diameter [pixel] | Separation force [%] | Smoothing filter Sigma | Method | Constrained channels | Detection sensitivity [%] | Donut width [pixel] |  |
| Advanced Morphology for Tissue | DAPI | 120 | 15/200 | 120-200 | 1,0 | Constrained Donut | Automatic | 120 | 1000 | Tonsil |
| Advanced Morphology for Tissue | DAPI | 200 | 20/200 | 130 | 1,0 | Constrained Donut | Automatic | 200 | 1000 | CRC |
| Advanced Morphology for Tissue | DAPI | 190 | 20/200 | 120 | 3,0 | Constrained Donut | Automatic | 200 | 1000 | CCC |
| Advanced Morphology for Tissue | DAPI | 110-120 | 20/200 | 65-85 | 1,0 | Constrained Donut | Automatic | 80-100 | 1000 | HCC |
| Advanced Morphology for Tissue | DAPI | 190 | 20/200 | 110 | 1,0 | Constrained Donut | Automatic | 200 | 1000 | PCa |
| Comments and recommendations | | | | | | | | | | |
|  | Choose DAPI channel with highest signal intensity and adjust the signal optimally before segmentation. | Depending on intensity of DAPI signal across the different cells in the sample. | Use a bigger range for including different cell types/ structures. | Loose cell composition 🡪 lower value, densely packed cell composition 🡪 higher value. | Depending on noise of the image: Higher value 🡪 reduced detail, nuclear shape smoothened. |  | Depending on the panel: Choose markers which are covering most cell types. E.g., CD2, CD3, CD38, CD44, CD45, CD8, Cytokeratin, HLA-ABC, HLA-DR, HLA-DR/DP/DQ, Podoplanin, Vimentin | Detection of brighter cells  🡪 lower value, more faint cells  🡪 higher value. | Choose the maximum (1000), the software is testing the size and automatically adjusts size of the cytoplasmic region. |  |

**Table S3: Advanced segmentation parameters for MACS iQ View.**
